# Integration of Metabonomics and Transcriptomics Reveals the Therapeutic Effects and Mechanisms of *Baoyuan* Decoction for Myocardial Ischemia

**DOI:** 10.3389/fphar.2018.00514

**Published:** 2018-05-23

**Authors:** Zhiyong Du, Zeliu Shu, Wei Lei, Chun Li, Kewu Zeng, Xiaoyu Guo, Mingbo Zhao, Pengfei Tu, Yong Jiang

**Affiliations:** ^1^State Key Laboratory of Natural and Biomimetic Drugs, School of Pharmaceutical Sciences, Peking University, Beijing, China; ^2^Modern Research Center for Traditional Chinese Medicine, School of Chinese Materia Medica, Beijing University of Chinese Medicine, Beijing, China

**Keywords:** myocardial ischemia, traditional Chinese medicine formula, *Baoyuan* decoction, pharmacodynamics, metabonomics, transcriptomics, multi-omics

## Abstract

Myocardial ischemia (MI) is an escalating public health care burden worldwide. *Baoyuan* decoction (BYD) is a traditional Chinese medicine formula with cardioprotective activity; however, its pharmacological characteristics and mechanisms are obscured. Herein, a multi-omics strategy via incorporating the metabonomics, transcriptomics, and pharmacodynamics was adopted to investigate the effects and molecular mechanisms of BYD for treating MI in a rat model of left anterior descending coronary artery (LADCA) ligation. The results indicated that BYD has a significantly cardioprotective role against MI by decreasing the infarct size, converting the echocardiographic abnormalities and myocardial enzyme markers, and reversing the serum metabolic disorders and myocardial transcriptional perturbations resulting from MI. Integrated bioinformatics analysis and literature reports constructed the interaction network based on the changes of the key MI targeted-metabolites and transcripts after BYD treatment and disclosed that the cardioprotection of BYD is mainly involved in the regulation of energy homeostasis, oxidative stress, apoptosis, inflammation, cardiac contractile dysfunction, and extracellular matrix remodeling. The results of histopathological examination, quantitative RT-PCR assay, cardiac energy synthesis, and serum antioxidant assessment complemented the multi-omics findings, and indicated the multi-pathway modulation mechanisms of BYD. Our investigation demonstrated that the multi-omics approach could achieve a complementary and verified view for the comprehensive evaluation of therapeutic effects and complex mechanisms of TCMF like BYD.

## Introduction

Myocardial ischemia is a disorder characterized by a critical decrease in the supply of myocardial oxygen and nutrients in the heart muscle as the result of a coronary artery obstruction. MI may damage cardiomyocytes and reduce cardiac function, thereby leading to myocardial infarction, myocardial fibrosis, and heart failure. Despite some advances have been made in the treatment of MI, the mortality rate caused by MI remains high (Shimokawa and Yasuda, 2008; Le Page and Prunier, 2015). Therefore, search for effective drugs that can ameliorate the MI extent are of substantial clinical and healthy values. In recent years, TCMFs have received substantial interest and increasing acceptance worldwide for the treatment of multitudinous diseases. For example, Compound Danshen Dropping Pills, a widely used TCMF in China, has been approved by the Food and Drug Administration (FDA) of United States for the stages II and III Investigational New Drug (IND) examinations (Sun et al., 2013).

In general, a TCMF is often composed of several medicinal herbs with complex chemical ingredients, and multiple targets and treatment functions (Leung et al., 2013). BYD, a well-known classic TCMF was initially archived in *Bo Ai Xin Jian* of the Ming dynasty, consists of a 6:2:2:1 ratio of astragalus root, ginseng, processed licorice, and cinnamon. BYD has the effects of tonifying *Qi*, reinforcing deficiency and warming *Yang*, and has been clinically used for the treatment of cardiovascular disease (CVDs), aplastic anemia, and chronic renal failure, etc. (Ma et al., 2016). Especially for the CVDs, BYD presented good therapeutic effects on angina, arrhythmias, chronic heart failure, and ischemic heart diseases. The main components found in BYD were disclosed to be ginsenosides, astragalosides, licorice saponins, and flavonoids (Ma et al., 2015, 2016, 2017). Notwithstanding that many active compounds have been identified from BYD and some of their potential effects in relation to the treatment of CVDs have been disclosed (Shu et al., 2016; Wan et al., 2017; Zhang et al., 2017), there still exists a challenge in clarification of the action mechanisms of BYD because of the complex interaction of its multi-components.

Systems biology is based on a series of omics technologies and provides a novel insight and approach in the field of TCMF research (Yan et al., 2015). The power of omics research has been proven to lead to breakthroughs in biomarker discovery and to provide insights into the underlying pathophysiology and new targets of intervention (Eichner et al., 2014). Nevertheless, to date, no single omics-platform is able to capture a complete overview of biological information in a comprehensive and systematic way. Therefore, an increasing number of studies have adopted multi-omics technique to obtain the integrated data sets, which can provide an improved understanding of the underlying biology and further insights into the complex mechanisms of the global system (Cavill et al., 2016).

Actually, TCMFs have many common characteristics with omics, such as the holistic, individual, and dynamic views; thus omics strategy may be a valuable technique for understanding TCMFs. However, the study of TCMFs by multi-omics is little. Herein, we proposed a multi-omics strategy via incorporating the metabonomics, transcriptomics, and pharmacodynamics to investigate the therapeutic effects and molecular mechanisms of TCMF. As a proof of concept, BYD was selected as a case and its effects and mechanisms for treating MI were illustrated by such a strategy. Potential biomarkers were identified to better understand the pivotal mechanisms of MI and the intrinsic linkages of the differentially expressed genes and metabolites were constructed to enhance the reliability of biomarkers for the diagnosis and management of MI, as well as to investigate the potential therapeutic effects and network mechanisms of BYD for the treatment of MI.

## Materials and Methods

### Chemicals and Reagents

Ultrapure water (18.2 MΩ) was prepared using a Milli-Q water purification system (Millipore, Billerica, MA, United States). Acetonitrile (HPLC grade) and methanol (HPLC grade) were purchased from Merck (Darmstadt, Germany). Formic acid (LC/MS grade) was purchased from Fisher Scientific (Spain). Leucine encephalin was supplied by Sigma-Aldrich (St. Louis, MO, United States). Cyclic AMP, cyclic GMP, retinoic acid, L-arginine, L-isoleucine, L-methionine, stearic acid, L-phenylalanine, L-valine, L-histidine, stearic acid, and uridine were purchased from Sigma-Aldrich (St. Louis, MO, United States). LysoPC (18:0) and LysoPC (17:0) were purchased from Avanti Lipids Polar, Inc. (Alabaster, AL, United States). Other chemicals were all analytical grade. FastQuant RT (With gDNase) was obtained from TianGen Biotech (Beijing, China). SYBR TransStart Green qPCR SuperMix was purchased from TransGen Biotech (Beijing, China). The positive control drug, *Ginkgo Biloba* extract 761 (EGb 761) was purchased from Dr. Willmar Schwabe GmbH & Co. KG.

### Preparation of BYD

All crude materials were collected from a TCM market (Anguo, Hebei, China), and authenticated by Prof. Pengfei Tu to be the roots of *Astragalus membranaceus* (Fisch.) Bunge var. *mongolicus* (Bunge) Hsiao, *Panax ginseng* C. A. Mey., prepared *Glycyrrhiza uralensis* Fisch, and the barks of *Cinnamomum cassia* Presl. All of the voucher specimens (PG-AG-20130312, GU-AG-20130312, AM-AG-20130312, and CC-AG-20130312) have been deposited at the Modern Research Center for Traditional Chinese Medicine, Peking University (Beijing, China). BYD was prepared by combining the crude astragalus roots (30 kg), ginseng (10 kg), processed liquorice (10 kg), and cinnamon (5 kg) at a ratio of 6:2:2:1. The mixed crude drugs were soaked with 550 L water for one night and subsequently refluxed for 2 h at 100°C three times. All filtered solutions were condensed in vacuo and subsequently freeze-dried to provide 16.5 kg BYD powders for experimental use. The lyophilized BYD powder was thoroughly dissolved in distillation water for the animal administration. The chemical profiling of BYD has been described in our previous report, and 36 representative primary components were accurately quantified for the QC (Ma et al., 2016).

### Animals and Ethics Statement

All animal studies followed the relevant national legislation and were approved by the Institutional Animal Care and Use Committee of Peking University Health Science Center. Ninety Sprague-Dawley rats (180–210 g, male) were purchased from the Animal Center of Peking University Health Science Center (Beijing, China) and were fed a certified standard diet and water. The temperature and humidity were set at 21–23°C and 40–60%, respectively. A 12 h light/dark cycle was used. All animals were acclimatized in the metabolism cages for 1 week prior to experiment procedure.

### Myocardial Ischemia Mode and Drug Administration

First, the widely used MI model produced by occlusion of the LADCA was employed to induce MI in rats (Ytrehus, 2006). Prior to the operation, the rats were fasted overnight with free access to water. Briefly, an anterior thoracotomy was performed under sterile conditions to open the pericardium and the heart was then rapidly exteriorized. The LADCA was ligated approximately 2–3 mm distal from its origin with the use of a 5-0 polypropylene suture. All experiment animals after operation were randomly divided into six groups (15 rats per group): (A) sham group (without coronary artery ligation), (B) MI group, (C) EGb 761 (positive drug)-treated group, (D) low dose of BYD-treated group, (E) middle dose of BYD-treated group, and (F) high dose of BYD-treated group.

After surgery, the rats in the groups A and B received the same volume of saline vehicle and the rats in the group C received EGb 761 at 100 mg/kg, an optimal effective dose that had been verified in a MI rat model (Li et al., 2015). The rats in the groups D, E, and F were administered BYD after MI induction at the doses of 365 mg/kg, 730 mg/kg, and 1460 mg/kg, respectively, which were calculated from the equivalent conversion by the body surface area between animals and human based on the recommended daily human dosage (Nair and Jacob, 2016) and the extraction percentage of BYD (∼30%). The drugs and vehicle were orally administered once per day for seven consecutive days. Fifty-six animals survived throughout the experiment, whereas 34 animals died after surgery or during the echocardiographic experiment and were excluded, including five animals in the A group, six animals in the B group, seven animals in the C group, five animals in the D group, five animals in the E group, and six animals in the F group.

### Echocardiographic Evaluation

To confirm the MI status and evaluate the efficacies of BYD, echocardiographic examinations were performed after consecutive drug administration for 7 days. All rats were anesthetized using sodium pentobarbital (60 mg/kg) and a Vevo770 ultrasound system (Visualsonics Inc., Toronto, ON, Canada) with a 17.5 MHz probe was employed for echocardiography. The sampling frequency in M-mode was 1000/s and the scanning speed was 50 - 100 mm/s. The probes were placed on the precordium and the detection was conducted from the section of ventricular bands. The LV parameters were obtained from 2-dimensional images and M-mode interrogation in the long-axis view. The LV FS and EF were recorded and the data were averaged over five consecutive cardiac cycles.

### Sample Collection

Under the deeply anesthetized condition after echocardiographic examinations, all rats were sacrificed for collecting the serum and heart tissue samples. Blood samples were collected from the abdominal aorta, and the serum samples were obtained by centrifuging the blood samples for 10 min at 3000 rpm and 4°C. Hearts were harvested immediately after blood collection. The serum and heart tissue samples were stored at -80°C for further analysis.

### Myocardial Infarct Size Measurement

The frozen heart was cut into six thick short-axis sections (1 mm per slice) from the apex toward the base of the heart. The slices were then incubated in TTC in pH 7.4 phosphate buffer solution for 30 min at 37°C, and then fixed in 4% paraformaldehyde for another 30 min. The TTC stained the normal tissue dark red while the infarct area remained grayish-white. The slices were photographed and the infarct size was determined as a ratio of the LV infarct area to the whole LV area by using the software of Image J software.

### Histology and TUNEL Assay

The heart tissue samples were fixed in 10% neutral-buffered formalin at room temperature for 24 h after a brief rinse with phosphate buffered saline (PBS), and the heart tissue specimens were subsequently embedded in paraffin. The paraffin sections, approximately 3 μm, were stained with routine H&E staining for histological examination. TUNEL staining was performed to detect and quantify the apoptotic cells according to the manufacturer’s protocol (Roche, Switzerland). Photographs were obtained with the use of a light microscope IX73 (Olympus, Germany) at 200 magnification.

### Biochemical Indicators

The serum concentrations of lactate dehydrogenase (LDH) and CK were measured via an ultraviolet spectrophotometer using commercial kits (Jiancheng Bioengineering Institute, Nanjing, China). For antioxidant assays, the T-AOC, SOD, MDA, and GSH-Px levels in the serum were spectrophotometrically measured using diagnostic kits (Jiancheng Bioengineering Institute, Nanjing, China) according to the manufacturer’s instructions. The contents of ATP and ADP in the heart tissue were assessed by ultraviolet spectrophotometer using commercial kits (Jiancheng Bioengineering Institute, Nanjing, China).

### Sample Preparation and LC-MS Conditions for Metabonomic Analysis

The serum samples were thawed at 4°C. A volume of 300 μL methanol was added to a 100 μL aliquot of serum to precipitate the proteins. After vortex-blending for 1 min and incubation on ice for 5 min, the mixture was centrifuged at 10000 rpm for 10 min at 4°C. The supernatant was transferred and diluted at a ratio of 1:1 with deionized water for LC-MS analysis. The QC sample was prepared by mixing an equal volume (10 μL) aliquot from each serum sample. The “pooled” QC sample was treated using the same procedure described above.

LC-MS analysis was performed on a Waters ACQUITY Ultra Performance Liquid Chromatography (UPLC) system coupled with a dual electrospray ionization probe and a Micromass Quadrupole (Q)-TOF micro Synapt High Definition Mass Spectrometer from Waters (Waters Corporation, Milford, MA, United States). Metabolite separation was achieved through an ACQUITY UPLC BEH C18 column (50 × 2.1 mm, 1.7 μm, Waters Corporation, Milford, MA, United States). The column was maintained at 30°C and the flow rate was 0.4 mL/min. A 2 μL aliquot of each sample was injected. The optimal mobile phase consisted of a linear gradient system of (A) 0.1% formic acid in water and (B) 0.1% formic acid in acetonitrile: 0-1.0 min, 3-20% B; 1.0-6.0 min, 20-60% B; 6.0-9.5 min, 60%B; 9.5-11.5 min, 60-90% B; 11.5-13.5 min, 90-100% B; 13.5-15.5 min, 100% B; 15.5-16.5 min, 100-3% B; 16.5-18.5 min, 3% B. In a pre-test of the QC samples, the metabolites were detected with greater ion intensities and a wider coverage in the positive mode than in the negative model, and the total ion chromatogram was more stable in the positive mode. Thus, only the positive mode was utilized for the following research. The parameters used for the mass detection were set as follows: the source temperature was set at 110°C; the desolvation gas temperature and desolvation gas flow were 350°C and 800 L/h, respectively; the capillary voltage was 3.0 kV; sampling cone voltage was 20 V; the extraction cone voltage was 1.0 V; the cone gas rate was set at 50 L/h and the collision energy was set at 5 eV. All analyses were acquired using a LockSpray interface to ensure the accuracy and reproducibility. Leucine-enkephalin (*m/z* 556.2771 [M+H]^+^) was used as the mass reference compound in the positive ion mode. Data was collected between *m/z* 50 - 1000 Da.

### Sample Repeatability and LC-MS System Stability

The stability and repeatability of the metabonomic method were assessed as follows: the extracts from six aliquots of the pooled serum samples were continuously injected to evaluate the repeatability. The RSDs of five extracted ions were less than 9.23% for the peak areas and less than 0.14% for the retention times, respectively. For evaluating the LC-MS system stability, the QC sample was injected every six real samples throughout the entire experiment, and the PCA score plot indicated that the QC samples were tightly clustered (Supplementary Figure 1). Moreover, the peak areas and retention times of the five extracted ions in the QC samples also showed good system stability. The RSDs of the five peaks were less than 7.25% for the peak areas and 0.79% for the retention times, respectively. These results indicated that the proposed method is robust for metabonomic analysis.

### Metabonomic Data Processing and Biomarker Identification

The raw data were obtained using the MarkerLynx Applications Manager Version 4.1 (Waters, Manchester, United Kingdom) for deconvolution, alignment, and data reduction to provide a list of data matrix. A MVA for the pareto-scaled metabolite data matrix was performed using SIMCA-P software (v14.0, Umetric, Umeå, Sweden). The quality of the MVA models was controlled by evaluating the *R*^2^ and *Q*^2^ values. An unsupervised PCA was employed to assess the quality, homogeneity, outlier identification, and dominating trends of the group separation inherent in the dataset. A supervised OPLS-DA was performed to discriminate the classes and identify the differentially expressed variables. The *p*-values of the cross-validated analysis of variance were used to assess the reliability of the OPLS-DA model and to test the inherent risk (overfitting) in supervised analysis (Eriksson et al., 2008).

A combination of the *S*-plot [an absolute *p* (corr) > 0.4 was used as the cutoff value] and the variable influence in the projection (VIP) plot (VIP > 1.5) from the OPLS-DA model was performed to identify the highest potential variables as biomarkers for MI diagnosis. The putative metabolites were derived by searching the exact molecular mass data from the redundant *m/z* peaks against the online HMDB^1^, METLIN^2^, and KEGG^3^ databases. A specific metabolite was sieved out when a difference between the observed and theoretical masses was less than 5 ppm, along with a higher i-Fit value by MarkerLynx. The molecular formula of the matched metabolite was further identified by the isotopic distribution measurement and the retention time and MS/MS fragmentation pattern of authentic standard. The identified biomarkers were subsequently confirmed by the *p*-values with a critical value of 0.05 from a Student’s *t*-test. The ROC curve was also performed to evaluate the accuracy of the metabolic biomarkers in distinguishing different groups using a web-based tool^4^. The sensitivity and specificity of the trade-offs were calculated for the selected metabolites by using the area under the ROC curve (AUC) in two options: the univariate ROC curve analysis and the multivariate ROC curve based model evaluation (Cutoff AUC area value was set at 0.8). Based on the validated biomarkers, an unsupervised Heatmap with an Euclidean distance measure (a logarithmic scale) was employed by using MetaboAnalyst 3.0^5^ to explore the correlation and visualize the hierarchical relationship among the sham, MI, and drug administration groups. A metabolic pathway analysis to facilitate the biological interpretation was performed using MetPA^5^ to identify the most relevant pathways.

### Transcript Profile Analysis

The myocardial tissues of the ischemic LV and the peri-ischemic rim (an approximately 1 mm rim of normal-appearing tissues) were removed, and the remaining tissues that consisted of non-ischemic LV were obtained for the transcriptional analysis. The total RNA was extracted from the myocardia from the sham (*n* = 3, randomly), MI model (*n* = 3, randomly), and high dose of BYD-treated MI (*n* = 3, randomly) groups using an RNAprep pure Tissue Kit (Beijing TianGen Biotech, China). The RNA integrity was analyzed on a 1% agarose gel. Beads that contained oligo (dT) were used to isolate the poly mRNA from the total RNA and then the rRNA was subsequently removed. The amplified library was sequenced on a HiSeq 2500 sequencing machine (Illumina, San Diego, CA, United States) with a 50 bp SE strategy. Using CASAVA software, the images generated by the Illumina sequencers were converted into raw reads by base-calling. To ensure the quality of the data for subsequent analyses, the clean reads were obtained from the raw data with three criteria to filter out the dirty raw reads: remove the reads with sequence adaptors; remove the low-quality reads that have more than 50% QA ≤ 19 bases; and remove the reads with more than 50% ‘N’ bases. The clean read sequences were annotated using TopHat software (v2.0.12) with the reference sequence of Rattus_norvegicus. Rnor_5.0.74. The reads that could be uniquely mapped to a gene were used to calculate the expression level. The gene expression data have been deposited in the ArrayExpress database at EMBL-EBI^6^ under accession number E-MTAB-6509.

### Transcriptomic Data Processing and Annotation of Differentially Expressed Genes

The gene expression level was expressed as the number of reads RPKM (Wagner et al., 2012) with Cufflinks software. The formula was defined as follows:

(1) $$\text{RPKM}\;=\;\frac{10^{6}{}^{*}\text{R}}{\text{NL}/10^{3}}$$

R was the number of reads uniquely mapped to the given gene; N was the number of reads uniquely mapped to all genes; and L was the total length of exons from the given gene.

The gene expression level in RPKM data (1 RPKM was set as a noise cut-off) was initially logarithmically transformed and pareto-scaled. An unsupervised PCA analysis was subsequently applied to visualize the general trends and cluster among the observations. The statistically altered genes in the sham and MI model groups of interest were extracted from the combined *S*- and VIP-plots in the OPLS-DA model, and together with an average cutoff of 1.5-fold changes. The quality and reliability of the OPLS-DA model were controlled by evaluating the *R*^2^, *Q*^2^, and CV-ANOVA *p*-values (Gennebäck et al., 2013; Cavill et al., 2016).

### Quantitative RT-PCR Assay

Differentially expressed genes were validated at the transcript level via quantitative RT-PCR (qRT-PCR). Total RNA was reverse-transcribed with a FastQuant RT (With gDNase) for cDNA synthesis and genomic DNA removal. The qPCRs were performed according to the instructions of the SYBR Green qPCR SuperMix and were conducted in triplicate using a Strategene Mx3005P system (Agilent Technologies, Santa Clara, CA, United States). The transcripts were amplified in one tube that contained 1 μg of cDNA and 0.1 μmol of each forward and reverse primer. PCR amplification was performed at 95°C for 10 min followed by 40 cycles at 95°C for 30 s, 54°C for 30 s, and 72°C for 60 s and a final extension at 95°C for 30 s, 55°C for 30 s, and 95°C for 30 s. Gene-specific primers were designed using the online primer design tool Primer-BLAST^7^ and were synthesized by Dingguo Changsheng Biotech Corporation (Beijing, China). The amplification lengths were between 80 and 150 bp (Supplementary Table 1). Glyceraldehyde-3-phosphate dehydrogenase (Gapdh) was selected as the endogenous control in this study. Standard deviations were calculated from three biological replicates and three PCR technical replicates. The specificity of the amplification was assessed using a dissociation curve analysis, and the relative abundance of the transcripts was determined using the Ct values. Significance was assessed with a one-way ANOVA (*p* < 0.05).

All functional analyses and enrichment for the differentially expressed genes verified by qRT-PCR were performed on a ClueGO 2.2.5 and CluePedia 1.2.5 within Cytoscape software 3.0. All GO terms were categorized in biological processes, molecular functions, cellular components; pathway analysis was categorized in the KEGG pathway and REACTOME pathway. The network was created with kappa statistics (significance was set at *p* < 0.05) and reflected the relationships between the functional terms based on the similarity of their associated expressed genes.

## Results

### Evaluation of MI Model and Therapeutic Efficacy of BYD

**FIGURE 1:**
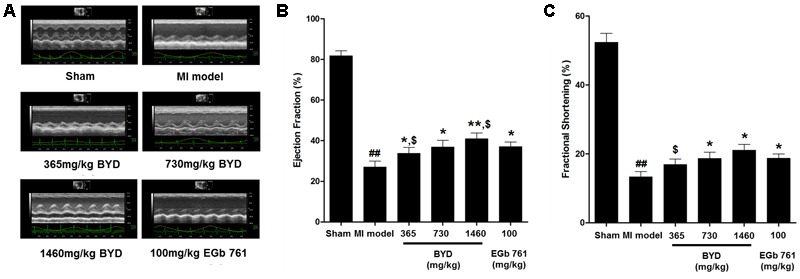
Echocardiographic assay. **(A)** Representative electrocardiogram recorded; **(B)** Values of EF (left ventricular ejection fraction); **(C)** Values of FS (left ventricular fraction shortening). Values are presented as means ± SD. Significance was assessed with a two-tailed Student’s *t-*test: ^##^*p* < 0.01 vs. the sham group; ^∗^*p* < 0.05 or ^∗∗^*p* < 0.01 vs. the MI group. ^$^*p* < 0.05 vs. the positive control group.

**FIGURE 2:**
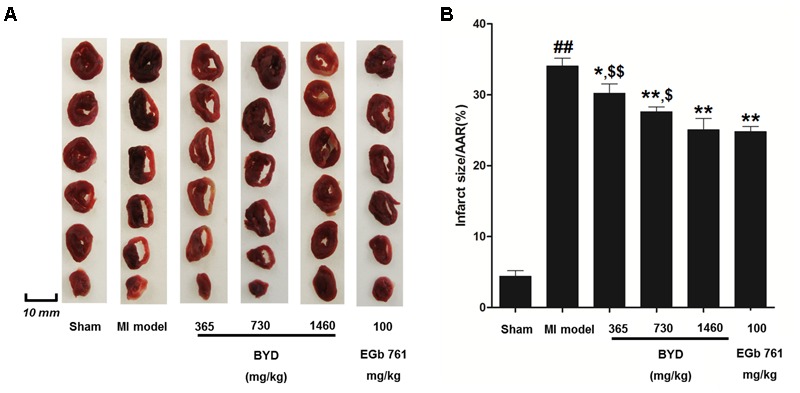
Effects of BYD on myocardial infarct size. **(A)** Representative images of the rat heart slices after TTC staining; **(B)** Quantitative analysis of infarct size. Values expressed as the mean ± SD, significance was assessed with a two-tailed Student’s *t-*test: ^##^*p* < 0.01 vs. the sham group; ^∗^*p* < 0.05 or ^∗∗^*p* < 0.01 vs. the MI group. ^$^*p* < 0.05 or ^$$^*p* < 0.01 vs. the positive control group. Abbreviations: area at risk, AAR.

**FIGURE 3:**
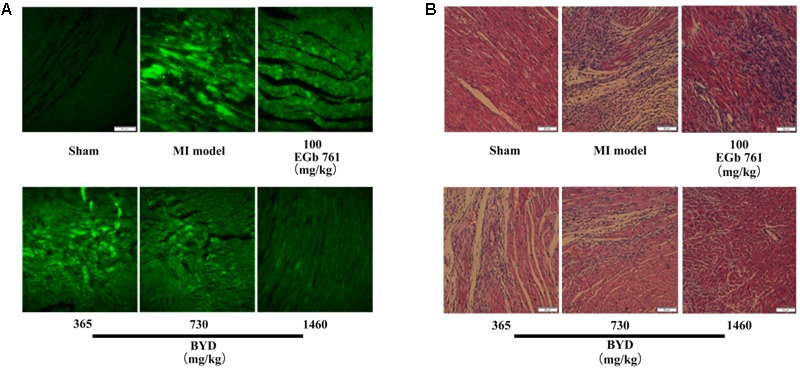
Histopathology and TUNEL assay. **(A)** Representative photomicrographs of cardiac tissue sections stained with H&E; **(B)** TUNEL assay of apoptotic cardiomyocytes after MI with BYD treatment; 200 × magnification.

**FIGURE 4:**
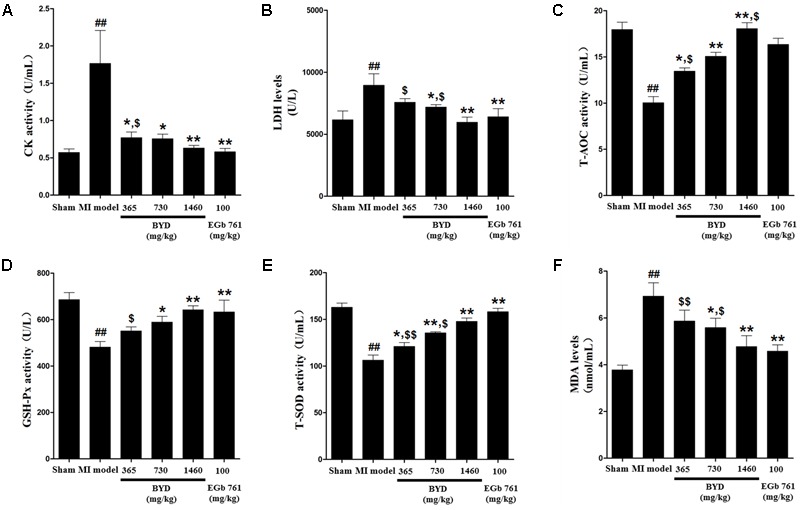
Effects of BYD on serum biochemical indicators in MI rats. **(A)** CK; **(B)** LDH; **(C)** T-AOC; **(D)** GSH-Px; **(E)** T-SOD; **(F)** MDA. Values expressed as the mean ± SD. Significance was assessed with a two-tailed Student’s *t-*test: ^##^*p* < 0.01 vs. the sham group; ^∗^*p* < 0.05 or ^∗∗^*p* < 0.01 vs. the MI group; ^$^*p* < 0.05 or ^$$^*p* < 0.01 vs. the positive control group.

**Figure 1** demonstrates the enhanced myocardial echo, thin-walled left ventricular, and significant decreases in the EF and FS for the MI rats compared with the sham rats. Moreover, the myocardial infarct sizes of the MI rats were significantly increased (**Figure 2**). After administration of the EGb 761 and the middle and high doses of BYD, the LV dilatations were substantially attenuated and the infarct sizes were remarkably reduced in the MI rats (**Figures 1, 2**). **Figure 3** provides the representative color photomicrographs of H&E- and TUNEL-stained heart tissue sections. The histopathological examination of the heart section of MI rats stained with H&E showed a widespread myocardial structure disorder, interstitial hemorrhage, leukocyte infiltration, myocardial swelling, and intercellular space widening with a myofibril focal degeneration. The TUNEL assay also indicated that remarkable apoptosis occurred in the myocardial tissues of the MI rats with an obvious green fluorescence compared with the sham group. The pathological abnormalities and extensive apoptosis were attenuated in the BYD-administered MI rats in a dose-dependent manner, and the effect of the group treated with a high dose of BYD was the most noticeable, and the effect of the group treated with a high dose of BYD was the most noticeable, exhibiting slighter pathological changes and lesser TUNEL positive cells in heart tissues compared to EGB 761-treated group. The MI rats also exhibited higher levels of LDH and CK, the cardiac markers in the serum than the sham group. While, oral administration of EGb 761 and BYD could significantly decrease their levels (**Figure 4**). These results confirmed that the MI model was successfully produced, and after pharmacological intervention with BYD, significant improvements in these parameters were observed in a dose-dependent manner, and the high-dosed BYD group demonstrated better activities in ameliorating the cardiac function and histological pathological changes compared with the positive control of EGb 761 group (**Figures 1, 3**). These findings suggest that BYD exerts a potential therapeutic effect against MI.

### Classification of the Serum Metabolic Profiles and Multivariate Biomarker Identification

**FIGURE 5:**
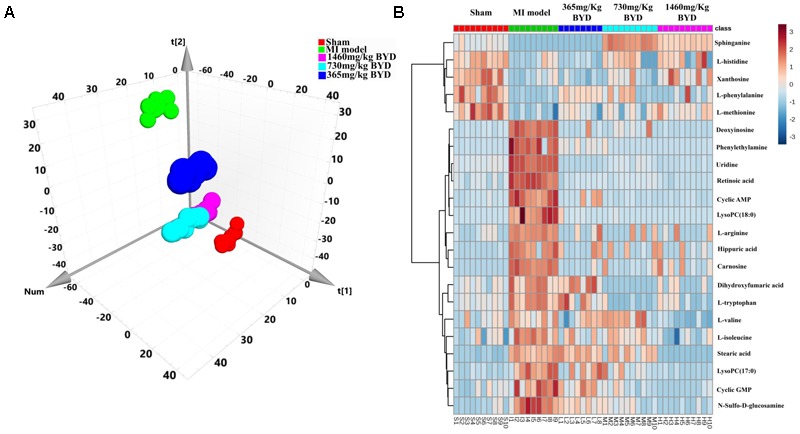
Efficacy classification models of the global metabolism and metabolic biomarkers between the BYD-treatment groups and MI group. **(A)** PCA 3D score plot (*R*^2^X = 0.574, *Q*^2^ = 0.459) of the sham group, MI group, and different doses of BYD-treated groups; **(B)** Heatmap of metabolic biomarker intensities in different groups.

An unsupervised PCA is the most frequently adopted approach to distinguish the classes in omics fields. To investigate the global metabolism variations and to evaluate the therapeutic effects of BYD on the MI rats, all observations were analyzed using PCA, which exhibited a clear classification in the score plot (*R*^2^X = 0.574, *Q*^2^ = 0.459; **Figure 5A**). The PCA 3D score plot indicated that the metabolic states of the MI and sham groups are readily separated and that all three BYD-dosed regimens are clearly separated from the MI group. The high and middle doses of the BYD-treated groups clustered together and located close to the sham group. Compared with the low-dosed group of BYD treatment, the high- and middle-dosed rats exhibited a better performance in the recovery of the MI-disturbed metabolic state and significantly altered the metabolic fingerprint of the MI serum.

Efficient diagnostic metabolite biomarkers are needed for the distinguishment between the diseased and non-diseased status. The unsupervised PCA scores plot indicated that the metabolic serum profiles of the MI rats deviate from those of the sham rats (*R*^2^X = 0.693, *Q*^2^ = 0.542; Supplementary Figure 2A). Furthermore, a remarkable separation between the MI and sham rats was also observed in the supervised OPLS-DA score plot (*R*^2^X = 0.491, *R*^2^Y = 0.99, *Q*^2^ = 0.978; Supplementary Figure 2B). The CV-ANOVA *p* value (5.5687 E^-38^) suggests that the OPLS-DA model is highly significant and implies non-overfitting. These results suggested that significant biochemical changes occurred in the MI procedure. Twenty-five ions that contributed to the separation of the MI and sham groups were selected and identified from the loading *S*- and VIP-value plots of the OPLS-DA (Supplementary Figure 2C). All these identified metabolites were furthered confirmed by the univariate ROC curve analyses (AUC > 0.8; Supplementary Figure 3) and Student’s *t*-tests (*p* < 0.05). Twenty-two metabolites were ultimately considered to exhibit the greatest sufficient utility and diagnostic accuracy for discrimination of the MI and sham groups and are listed in Supplementary Table 2. Moreover, the ROC curve-based model evaluation was established, and the AUC value obtained in this analysis is 0.948, indicating a good diagnostic accuracy for evaluation of the potential application of these differential metabolites for the MI diagnosis (Supplementary Figure 4A).

### Metabonomic Evaluation of BYD and Pathway Analysis Based on Metabolic Biomarkers

To further investigate the recovery condition of the 22 diagnostic bio-candidates by BYD, the relative peak areas between the three BYD-dosed groups and the MI group were tested via univariate ROC curve analyses and Student’s *t*-tests (Supplementary Table 2). With the exception of L-histidine, all other biomarkers were significantly reversed in the high BYD-dosed group. For the middle dosed group, with the exception of L-histidine, L-methionine, stearic acid, and xanthosine, the other 18 biomarkers could be restored to a certain degree. However, for the low BYD-dosed group, only 13 biomarkers were improved comparing with the MI rats. In addition, the AUC values of the established model evaluation by these 22 altered metabolites presented ideal values of 0.928, 0.876, and 0.646 for the high, middle, and low BYD-dosed groups versus the MI group, respectively (Supplementary Figures 4B–D). Similarly, the intensities of 22 biomarkers in the high and middle BYD-dosed and sham groups exhibited similar patterns, which are distinct from the MI and low BYD-dosed groups, as shown in the Heatmap visualization (**Figure 5B**). Using MetPA, the ingenuity network analysis was employed to identify the most relevant pathways for MI and to evaluate the impact on these pathways after different BYD-dosed treatments (Supplementary Table 3 and Supplementary Figure 5). It is noteworthy that the pathway enrichment and topology analysis indicated that MI targeted-amino acid metabolisms were significantly improved after BYD treatment.

### Classification of the Transcriptomic Phenotypes and Identification of the Altered Transcripts

**FIGURE 6:**
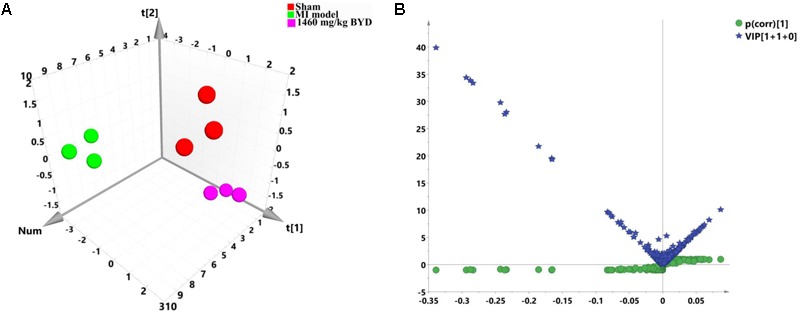
Pattern analysis of the data from the transcriptional profiles of myocardial tissues. **(A)** PCA 3D score plot (*R*^2^X = 0.817, *Q*^2^ = 0.675) of the sham group, MI group, and high dose of BYD-treated group; **(B)** A combination plot of *S*-plot and VIP values.

A non-target global gene expression analysis was employed to explore how MI altered the myocardial mRNA expression, as well as to evaluate the intervention of the high dosed-BYD treatment on the MI rats based on its impressive efficacy shown in the metabonomic and pharmacodynamic studies. As presented in **Figure 6A**, significant discrimination was achieved with the PCA model (*R*^2^X = 0.817, *Q*^2^ = 0.675). The PCA 3D score plot demonstrated a division of the MI and high BYD-dosed groups into two distinct clusters with a clear separation; moreover, the high BYD-dosed and sham groups fell into the same region, which indicated that BYD treatment can reverse the myocardial transcriptomic profiles of MI rats to the normal status.

In order to search for differentially expressed genes in MI rats, a PCA model was initially adopted to analyze the transcriptomic profiles of the MI and sham groups, and a satisfactory discriminative model is presented in the PCA scatter plot (*R*^2^X = 0.884, *Q*^2^ = 0.676; Supplementary Figure 6A). Next, an OPLS-DA model (*R*^2^X = 0.879, *R*^2^Y = 0.998, *Q*^2^ = 0.978; Supplementary Figure 6B) along with a CV-ANOVA *p* value (0.00033) was subsequently constructed to maximize the separation and identify the differentially expressed genes that contributed to the separation of the transcriptomic patterns of the MI and sham groups. Furthermore, 35 differentially expressed transcripts were selected by combining the *S*- and VIP plots in OPLS-DA for the MI diagnosis (**Figure 6B**). All expression levels of 35 candidate transcripts (Supplementary Table 1) were validated at the transcript level by qRT-PCR. The qRT-PCR results statistically indicated that the MI led to the increases of 11 gene expressions (Ankrd1, Nppa, Sparc, Mgp, Myh7, Crsp3, Col3a1, Tmsb4x, Fth1, Hba-a2, and Tnnc1) and the decreases of 18 gene expressions (mitochondrial DNAs, Myh6, Fam111a, Gbp1, Phyh, Myl3, Myl2, and Cryab) (Supplementary Figure 7).

### Therapeutic Evaluation of BYD and Bioinformatics Analysis Based on Altered Transcripts

**FIGURE 7:**
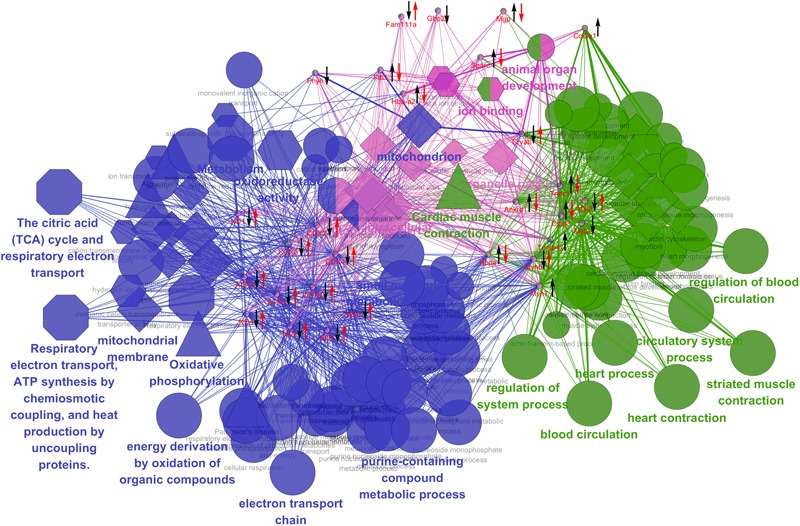
Grouping of network based on functionally enriched GO terms and pathways. Functionally grouped network of enriched categories was generated for the differentially expressed genes and their regulators using ClueGO and CluePedia analysis. GO terms and pathways are represented as nodes, and the node size represents the term enrichment significance. Functionally grouped networks are linked to their biological function, where only the most significant term in the group is emphatically labeled. Biological process is marked with an ellipse; Molecular function is marked with a hexagon; Cellular component is marked with a diamond; KEGG pathway in triangle; REACTOME pathway in an octagon. The left black arrow “↓” or “↑” indicates a decreased or increased level, respectively, of the altered expressed candidates in the MI group compared with the sham group; the right red arrow “↓” or “↑” indicates a decreased or increased level, respectively, of the candidate genes in the high dose of BYD-treated group compared with the MI group.

The relative abundances of these MI target transcripts were further used to investigate the therapeutic effects of BYD on MI via the qRT-PCR assays. With the exception of Gbp1, Phyh, Myl2, Myh7, Col3a1, and Tmsb4x, the other 23 transcripts in the MI rats were significantly reversed after BYD treatment (Supplementary Figure 7). We then functionally associated these differentially expressed genes in MI rats and the most significant GO terms and pathways visually by ClueGO and CluePedia to explain the biological processes of the BYD effects (**Figure 7**). In general, the affected annotation covered by the differentially expressed genes elucidated sophisticated biological process categories, such as oxidative phosphorylation, cardiac muscle contraction, and cellular process. Moreover, the mitochondrial energy metabolism related categories were found to be located at the central hub node among the functional groups.

### Interpretation of the Integrated Metabonomics and Transcriptomics Information

**FIGURE 8:**
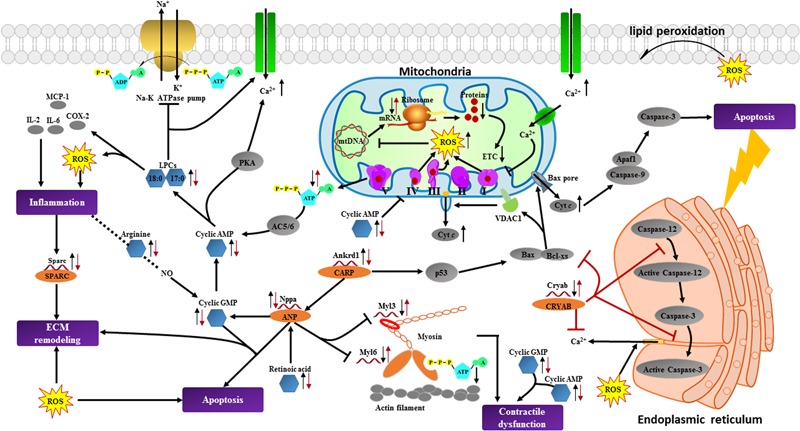
Summary of cardioprotective effects of BYD on the network of vital metabolic biomarkers and cardiac transcripts in MI rats. The left black arrow “↓” or “↑” indicates a decreased or increased level, respectively, of the differentially expressed alterations in the MI group compared with the sham group; the right red arrow “↓” or “↑” indicates a decreased or increased level, respectively, of the alterations in the high dose of BYD treated group compared with the MI group. Cytc, Cytochrome *c*; VDAC1, Voltage dependent anion channel protein 1; AC, adenylyl cyclase; PKA, protein kinase A; Apaf1, apoptotic protease activating factor-1; CARP, cardiac ankyrin repeat protein. Significantly altered metabolites found in metabonomic results are marked in blue hexagons; differentially expressed transcripts identified in transcriptomics and corresponding proteins are marked in red single helixes and orange ellipses; potentially related and non-detected proteins are marked in gray ellipses; potential biological processes are marked in purple rectangles.

In order to obtain a comprehensive view of the complex mechanisms of BYD, an integration of the metabonomic and transcriptomic data was performed. In contrast to the easier integration of transcriptomics and proteomics, it is not possible to link metabolites directly to transcripts because there are no direct associations between them (Cavill et al., 2016); thus, we attempted to construct an integrated network of the significantly altered metabolites and transcripts via their associated proteins to explore the potential intrinsic connections among them. The corresponding proteins for the metabolic biomarkers were initially collected by searching the HMDB and 453 proteins were obtained; these proteins together with the candidate transcripts were categorized according to their main biological functions and interactions. The association network using STRING (a search tool for the retrieval of interacting genes/proteins) was constructed to facilitate the access to the BYD-triggered differentially expressed metabolites and genes in a visual synergistic interaction (Supplementary Figure 9). Through examination of the statistical entries for the functional terms (Supplementary Table 4), we determined that the main biological processes significantly affected by BYD involve energy metabolism, oxidative stress, apoptosis, cardiac muscle contraction, remodeling of the ECM, and inflammation. The major interaction network of the metabolic biomarkers and the altered cardiac transcripts is depicted in **Figure 8**.

### Therapeutic Effects of BYD on the Energy Synthesis and Oxidative Stress Induced by MI

Integrated bioinformatics analysis found that energy metabolism is a central concatenation hub of the transcriptomics and metabolomics data, thus several methods were adopted to validate this statistical result. The mRNA expression levels of mitochondrial DNAs (e.g., ATP6, ATP8, ND1, ND2, ND3, and CYTB, etc.) were detected by the qRT-PCR, and the results revealed that they were significantly decreased in MI rats and increased in BYD rats (Supplementary Figure 7). This implied that BYD might potentially increase ATP synthesis via oxidative phosphorylation and decrease accumulation of ROS generated from ETC complexes (Supplementary Figure 8A).

In order to support the above assumption, the ratio of ATP/ADP was determined to assess the capability of ATP generation in heart tissues (Han et al., 2017). Compared with the sham group, the ratio of ATP/ADP was significantly decreased in the MI group; while after BYD treatment, the ATP/ADP ratio was obviously increased (Supplementary Figure 8B). In addition, the MI rats also exhibited a higher serum level of MDA and lower activities of T-SOD, T-AOC, and GSH-Px than those in the sham rats. Whereas, oral administration of BYD could significantly ameliorate these serum biochemical indicators and potentially enhance the antioxidant capability of scavenging ROS in MI rats (**Figure 4**). The positive drug EGb 761 has a significant antioxidant property as a free radical scavenger (Kanter, 2011). We found that the high dose of BYD-treated group showed a better antioxidant activity in increasing the activity of T-AOC compared with the EGb 761-treated group. All these findings indicated that BYD treatment might ameliorate the dysfunction of energy metabolism and oxidative stress responses.

## Discussion

For MI specific studies, several clinical trials and animal models have been used to identify the differential alterations, disclose the altered pathways, and monitor the efficacy of the drugs; and a considerable number of molecules highly correlated with MI were identified as potential biomarkers by metabolomics or transcriptomics methods. However, under different conditions or strategies, the identified biomarkers are different; thus, the discovery of novel and validated biomarkers remains ongoing (Griffin et al., 2011; Bodi et al., 2012; Muehlschlegel et al., 2015; Guo et al., 2016). Moreover, the single metabonomics or single transcriptomics strategy cannot give a comprehensive and systematic insight of the pathophysiological processes in MI. TCMFs have been used for 100s of years, but their underlying therapeutic mechanisms remain poorly understood. It is a challenge to illustrate the action mechanisms of TCMF as a result of the unknown mutually synergistic actions of therein complex components. Both TCMFs and omics theories have holistic, individual, and dynamic views in common, and the omics technique has thus been accepted as a valuable technique for understanding TCMFs from a holistic and comprehensive approach (Qiu, 2007; Wong et al., 2016).

In the present study, our findings indicated that BYD, a specific TCMF for treating CVDs, could substantially improve the echocardiographic, pathological, and biochemical perturbations in the MI rats. EGb 761, a commercially available herbal extract from *Ginkgo biloba* leaves has been investigated in preclinical studies for treating MI and reperfusion in European countries. The cardioprotective effects of EGb 761 are based on its roles in promoting vasodilation, inhibiting cardiac fibrosis, and enhancing antioxidant activity (Kanter, 2011; Li et al., 2015). Compared to EGb 761-treated group, the high dose of BYD-treated group showed better effects on improvement of the MI-induced cardiac dysfunction and remodeling and total antioxidant activity, indicating that the unique advantages of herbs combinations and the synergistic actions of multiple ingredients in TCMF.

Furthermore, we integrated the untargeted metabonomics and transcriptomics techniques to obtain MI-related altered molecules and evaluate the therapeutic effects of BYD. We combined the integrated analysis of systems biology with literature data in order to obtain a comprehensive understanding of the pathophysiological processes of MI and the potential mechanisms of BYD’s actions. Our results indicated that BYD may have a remarkable role in regulation of energy metabolism which is closely related to its function of tonifying *Qi* and warming *Yang*, as well as the other potential properties of anti-oxidation, anti-apoptosis, anti-inflammation, and anti-myocardial remodeling through modulation of multi-pathways.

The qRT-PCR assays (Supplementary Figure 7) indicate that the expressions of mtDNAs that encode the ETC complexes decrease in the MI rats. The impaired mtDNAs expressions trigger the reduction of ATP generation and ROS generation during MI (Lenaz, 2001; Murphy, 2009; Chen and Zweier, 2014). Amino acids are essential for protein synthesis and are also utilized as substrates to generate energy. For example, isoleucine can be catabolized to generate succinyl-CoA, and tryptophan can be transformed into pyruvate and fumarate to enter into Krebs cycle (Knapp et al., 1986; Nemutlu et al., 2015). Despite an increased demand for amino acids in the MI heart, there is evidence of amino acids accumulation in the failing myocardium, and the increased levels of amino acids are associated with the abnormal energy homeostasis, superoxide production accumulation, and mitochondrial respiration disorder (Stanley, 2004; Sun et al., 2016; Wende et al., 2017). In our findings, the serum levels of arginine, tryptophan, isoleucine, phenylalanine, and histidine were all elevated in MI rats. The perturbation of amino acids and the impaired mtDNA expressions indicated the mitochondria energy dysfunction after MI.

After BYD administration, both amino acids and transcripts encoded ETC were significantly reversed; moreover, treatment with BYD significantly prevented the decrease of ATP/ADP ratio caused by MI. In our following identification of the targets for cardioprotection of BYD based on “target fishing” strategy, the most binding proteins identified by proteomics were found to be located in mitochondria (e.g., Aco2, Sucla2, Atp5c1, and Atp1a1, etc.), and the KEGG analysis revealed that these target proteins were mainly associated with Krebs cycle and amino acid metabolism signaling pathways (Wan et al., 2017). This result supported our above deduction that the cardioprotection of BYD is closely related to the mitochondrial energy metabolism and amino acid metabolism.

ROS is mainly generated from the leak of electrons, which may be induced by the inhibition and oxidative injury of complexes I and III in the ETC (Lenaz, 2001; Murphy, 2009; Chen and Zweier, 2014). Excessive ROS may further cause mtDNA damage and further decrease mtDNA expression (Ide et al., 2001). Methionine was reported to have a significant effect on myocardial antioxidant enzyme activities, especially on the activity of GSH-Px (Seneviratne et al., 1999), and excessive arginine is thought to induce oxidative stress via NO production (Delwing et al., 2008). LysoPCs induce the production of mitochondrial ROS and increase the intracellular Ca^2+^ by inhibiting the Na^+^-K^+^ ATPase pump (Prielipp et al., 1995; Li et al., 2016), and the accumulation of LysoPCs may contribute to the increased concentration of cyclic AMP (Ahumada et al., 1979). In addition, the ischemic condition may also result in a cyclic AMP-mediated inhibition of the ETC complex IV activity and an increase of the ROS level (Prabu et al., 2006). LysoPCs also play an important role in a series of inflammatory processes, such as the upregulation of interleukins, cyclooxygenase-2, and monocyte chemoattractant protein-1 (Matsumoto et al., 2007).

Compared with the MI rats, the expressions of methionine and mtDNAs encoded ETC complexes were increased, and the serum levels of arginine, LysoPCs, and cyclic AMP were decreased in the BYD group. In addition, the enhanced biochemical levels of T-AOC, SOD, and GSH-Px (**Figure 4** and Supplementary Figure 8A), and the significantly reduced inflammatory infiltration in histopathological examination after BYD treatment indicated that the potentially antioxidant capability of scavenging ROS and anti-inflammatory effect were significantly improved after BYD treatment. We used an *in vitro* NO inhibition model to screen the anti-inflammatory effect of BYD and its active components, and the results showed that ginsenoside Rk1 and resokaempferol are the main anti-inflammatory components of BYD, and their mechanism is via blocking the activation of NF-kappa B and the JAK2/STAT 3 pathway (Yu et al., 2016, 2017). All these findings suggest that BYD could partially obstruct the oxidative stress and reduce inflammation in MI rats.

The accentuated ROS generation and inflammation also play important roles in the fibroblast activation and ECM synthesis (Chen and Frangogiannis, 2013). Based on the abnormal remodeling of the ECM, extensive fibrosis is the most common phenomenon observed in MI (Jellis et al., 2010). Fibroblasts may actively turn into myofibroblasts and the latter mainly participate in the synthesis of ECM components to replace the necrotic cardiomyocytes after MI (Squires et al., 2005; Jellis et al., 2010). In the present study, several alterations associated with ECM remodeling were up-expressed in the MI rats (Supplementary Figure 10). Secreted protein acidic and rich in cysteine (SPARC) is encoded by the Sparc gene. Previous studies revealed that Sparc was substantially upregulated in MI (Frangogiannis, 2015), which was a response to injury and inflammatory reactions (McCurdy et al., 2010; Unsold et al., 2014). Sparc production following MI may also accelerate ventricular dilation and myocardial dysfunction (Bradshaw, 2016). A previous study indicated that the expressions of Mgp in cardiomyocytes and fibroblasts were rapidly upregulated in response to cardiac overload (Mustonen et al., 2009). Nppa has also previously been reported to be related to an increased expression in fibroblasts in response to the ligation model, which corresponds to the transition of fibroblasts to myofibroblasts (Cameron et al., 2000). Retinoic acid was reported to be increased during the remodel after MI (Bilbija et al., 2012) and it could stimulate the expression of Nppa gene (Manolescu et al., 2014). BYD postcondition could restrain the expressions of Sparc, Mgp, Nppa, and retinoic acid in the MI rats, leading to the amelioration of fibrosis. This was observed from the reduced degree of the fibrotic area and the lesser vacuolar change in the heart tissue sections of the BYD-administrated rats compared with those of the MI rats (**Figure 3**).

Dysfunctional mitochondria also trigger cardiomyocyte apoptosis by impairing the ETC, releasing cytochrome *c*, activating caspases, and altering the cellular redox potential via massive ROS accumulation (Chen and Zweier, 2014). In the present study, we also found abnormal expression of several molecules that associated with apoptosis. Nppa and cyclic GMP analogs have been reported to induce apoptosis (Wu et al., 1997; Chiche et al., 1998; Suenobu et al., 1999). Cyclic GMP is synthesized by soluble guanylate cyclase in response to NO, and the synthesis of cyclic GMP may be activated by Nppa (Pilz and Casteel, 2003). Ankrd1 encodes the cardiac ankyrin repeat protein, and the overexpression of Ankrd1 has been reported to exacerbate cardiomyocyte apoptosis by promoting p53 activation and mitochondrial dysfunction (Shen et al., 2015). Moreover, Ankrd1 has been reported to induce the increase of Nppa expression (Wei et al., 2009). α-Crystallin B encoded by the Cryab gene plays a key role in cardiomyocytes apoptosis through interfering the activation of caspase-3 and caspase-12, sequestering Bax and Bcl-XS from the cytosol, and preventing their translocation into mitochondria to block cytochrome *c* release (Ganguly et al., 2014).

The expression levels of Nppa and Ankrd1 transcripts, and metabolite cyclic GMP were increased, and the expression level of Cryab was decreased in the MI rats. However, after the BYD intervention, the expression levels of Ankrd1, Nppa, and cyclic GMP significantly decreased, and the level of Cryab was significantly increased in the MI rats after BYD treatment. Moreover, we also found that BYD treatment could up-regulate the expressions of Cryab and its upstream proteins (e.g., MEKK6, P38, MAPKAP-K2) in the heart tissues of MI rats (Zhang et al., 2017). Microscopic analyses of the H&E- and TUNEL-stained results of heart tissue sections (**Figure 3**) indicated that a substantial decrease in myocardial necrosis and apoptosis after BYD treatment compared with the MI group. We used an OGD/R-induced cardiomyocyte apoptosis model to evaluate the protection effect of BYD and to search for its active components, and the results showed that BYD and 17 active single components have the anti-apoptosis activities, and the virtual targets screening of the active components by the PubChem BioAssay database showed that the potentially cardioprotective mechanisms are related to oxidative stress pathway, mitochondrial protection, anti-apoptosis, etc. (Shu et al., 2016).

Functionally systolic dysfunction refers to an adaptation in response to MI, which is characterized by LV dilation and contractile dysfunction (Ahmad et al., 2014). The echocardiography results (**Figure 1**) consistently demonstrate a marked LV dilation and a contractile impairment in the MI group. In addition, several biomarkers associated with cardiac contractility dysfunction were significantly changed in MI rats after BYD treatment. For instance, Myh6 gene encoding *α*-myosin heavy chain was markedly decreased in the transcriptional level of MI rats, and the decreased Myh6 expression may be a compensatory action during energetic change after MI (James et al., 2010). Myl3 gene encoding the myosin light chain may positively regulate the ATPase activity. The decreased expression of Myl3 in MI rats may lead to an energy obstruction to the myosin contraction system (Chen et al., 2008). Ankrd1 also acts as a negative regulator of cardiac gene expressions, (Bogomolovas et al., 2015) and the up-regulation of Ankrd1 expression could reduce the cardiac contractility by depressing the myosin genes expression (Jeyaseelan et al., 1997; Zolk et al., 2002). Both cyclic GMP and cyclic AMP are involved in the regulation of cardiac contractility (Goetz et al., 2014; Zoccarato et al., 2015), and high cyclic GMP concentrations may cross-activate the production of cyclic AMP (Pilz and Casteel, 2003). BYD administration could significantly elevate the expressions of Myh6 and Myl3 and suppressed the expressed levels of Ankrd1 and the serum levels of cyclic GMP and cyclic AMP, compared with the MI rats. These findings suggested that BYD may improve the cardiac contractile dysfunction by reversing the related biomarkers.

The present study and our previous researches (Shu et al., 2016; Yu et al., 2016, 2017; Wan et al., 2017) have proved that BYD and the active ingredients therein have multiple potential therapeutic properties against MI. Of particularly, the multi-omics results indicated that BYD could significantly regulate transcripts encoded ETC complexes and reverse the energy substrates and the oxidative stress related-metabolites, which were further supported by the pharmacodynamics effects of promoting the cardiac ATP generation and enhancing the serum antioxidation capacity. Amelioration of mitochondrial energy metabolism could also improve cardiac contraction and decrease the accumulation of ROS, which stimulates the downstream pathological pathways activation. Moreover, BYD also have a potential role against cardiac apoptosis, inflammation, and myocardial remodeling through ameliorating the expression of the key related molecules, such as Ankrd1, Nppa, cyclic GMP, cyclic AMP, and LysoPCs and reinstating their biological turbulences. The future in-depth mechanism investigations and verifications of these pathways are necessary to further decipher the complex and synergistic mechanisms of BYD.

## Conclusion

Overall, in this paper, a proper experimental method utilizing the cutting edge “multi-omics” strategy via incorporating metabonomics, transcriptomics, and pharmacodynamics, was used to investigate the effects and molecular mechanisms of BYD for treating MI in rats. The results demonstrate that BYD has the significant cardioprotective effect against MI via improvement of the pathological perturbations and amelioration of the metabolic and transcriptional disturbances and molecular biology parameters in MI rats. The coordinated changes in altered metabolites are tightly related to the differential transcripts, which give us a more accurate interaction network of the pathological and biochemical responses for MI stimulation and BYD therapy. From a holistic view, the main pathways modulated by BYD include energy metabolism, oxidative stress, apoptosis, cardiac contractile dysfunction, etc., and the energy metabolism is the vital hub among them. BYD realized the cardioprotection via the regulating the energy homeostasis and inhibiting the downstream oxidative stress responses, apoptosis, and some other pathologically biological disturbances. These findings indicate the comprehensive and synergistic mechanisms of BYD, and prove that the multi-omics approach could be an efficient means for the complex mechanism evaluation of TCMF like BYD.

## Author Contributions

YJ and PT designed the research. ZS, KZ, and CL performed the animal experiments and pharmacodynamic studies. WL, XG, and MZ performed the mass spectrometry and data analysis. ZD performed the transcriptional data analysis and related validation, and the statistical tests and bioinformatics analysis. ZD, ZS, and WL drafted the manuscript, and YJ finalized the writing. All authors read and approved the final manuscript.

## Conflict of Interest Statement

The authors declare that the research was conducted in the absence of any commercial or financial relationships that could be construed as a potential conflict of interest. The reviewer JC declared a shared affiliation, though no other collaboration, with one of the authors CL to the handling Editor.

## Footnotes

1 http://www.hmdb.ca/

2 http://metlin.scripps.edu/

3 www.genome.jp/kegg/

4 http://www.roccet.ca

5 http://www.metaboanalyst.ca/

6 www.ebi.ac.uk/arrayexpress

7 http://www.ncbi.nlm.nih.gov/tools/primer-blast/

## Abbreviations

ADP: adenosine diphosphate
ANP: atrial natriuretic peptide
ATP: adenosine triphosphate
AUC: area under curve
BYD: *Baoyuan* decoction
CK: creatine kinase
Cyclic AMP: cyclic adenosine monophosphate
Cyclic GMP: cyclic guanosine monophosphate
ECM: extracellular matrix
EF: ejection fraction
ETC: electron transport chain
FS: fractional shortening
GO: gene ontology
GSH-Px: glutathione peroxidase
H&E: hematoxylin and eosin
JAK2: janus kinase 2
LADCA: left anterior descending coronary artery
LDH: lactatedehydrogenase
LV: left ventricle
LysoPC: Lysophosphatidylcholine
MDA: malondialdehyde
MI: myocardial ischemia
mtDNA: mitochondrial DNA
MVA: multivariate statistical analysis
NADH: nicotinamide adenine dinucleotide
NO: nitric oxide
OGD/R: oxygen-glucose deprivation/recovery
OPLS-DA: orthogonal partial least squares discriminate analysis
PARP: poly ADP-ribose polymerase
PCA: principal component analysis
QC: quality control
qRT-PCR: quantitative real time PCR
ROC: receiver operating characteristic
ROS: reactive oxidative species
RPKM: per kilobase of exon region per million mapped reads
RSDs: relative standard derivations
SOD: superoxide dismutase
STAT 3: signal transducer and activator of transcription 3
T-AOC: total antioxidant capacity
TCMF: traditional Chinese medicine formula
TTC: 1% 2,3,5-triphenyltetrazolium chloride
TUNEL: terminal deoxynucleotidyl transferase-mediated dUTP nick-end labeling
VIP: variable influence in projection
