# Supplementary material for: Integration of Metabonomics and Transcriptomics Reveals the Therapeutic Effects and Mechanisms of Baoyuan Decoction for Myocardial Ischemia
Source: Front Pharmacol. 2018 May 23;9:514. doi: 10.3389/fphar.2018.00514 (PMC5974172; doi:10.3389/fphar.2018.00514)
Supplement: Supplementary file 1 [file Data_Sheet_1.docx]

***Supplementary Material***

**Integration of metabonomics and transcriptomics reveals the therapeutic effects and mechanisms of *Baoyuan Decoction* for myocardial ischemia**

***Zhiyong Du ^1#^, Zeliu Shu ^1#^, Wei Lei ^1#^, Chun Li ^2^, Kewu Zeng ^1^, Xiaoyu Guo ^1^, Mingbo Zhao ^1^, Pengfei Tu ^1^ and Yong Jiang ^1*^***

*^1^* *State Key Laboratory of Natural and Biomimetic Drugs, School of Pharmaceutical Sciences, Peking University, Beijing 100191, China, ^2^ Modern Research Center for Traditional Chinese Medicine, School of Chinese Materia Medica, Beijing University of Chinese Medicine, Beijing 100029, China*

^#^ These authors contributed equally to this work.

*** Correspondence:***Yong Jiang*

*yongjiang@bjmu.edu.cn*

# Supplementary Figures and Tables

**Supplementary Figure 1.** PCA score plot of the QC samples and tested samples. The QC samples are marked in in red circles, and the tested samples are marked in black triangles.

**Supplementary Figure 2.** Pattern analysis of the UPLC/TOF-MS data of serum samples from the sham and MI groups.

**Supplementary Figure 3.** Univariate ROC curve analysis for individual metabolic biomarkers of MI.

**Supplementary Figure 4.** ROC curve-based model evaluation.

**Supplementary Figure 5.** Summary of the metabolic pathway enrichment analysis in MetaboAnalyst.

**Supplementary Figure 6.** Pattern analysis of data from the transcriptional profiles of myocardial tissues.

**Supplementary Figure 7.** Effects of BYD on the mRNA expressions of the significantly altered genes in MI rat heart tissues by qRT-PCR assay.

**Supplementary Figure 8.** Effects of BYD on energy metabolism in the rat myocardium subjected to MI.

**Supplementary Figure 9.** Interaction analysis of metabolic biomarkers related- proteins and differentially expressed transcripts.

**Supplementary Figure 10.** Effects of BYD on extracellular matrix remodeling after MI.

**Supplementary Table 1.** DNA oligonucleotide primer sequences used for qRT-PCR analysis of differentially expressed genes.

**Supplementary Table 2.** Identification of significantly differential metabolites in rat serum.

**Supplementary Table 3.** Results from ingenuity pathway analysis with MetPA.

**1.1 Supplementary Figures**

**Supplementary Figure 1.** PCA score plot of the QC and tested samples. The QC samples are marked in in red circles, and the tested samples are marked in black triangles.

**A**

**B**


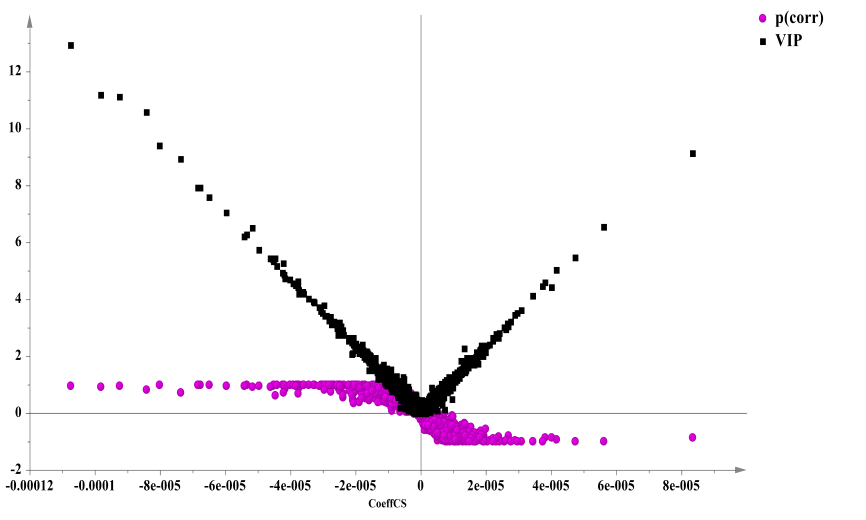


**C**

**Supplementary Figure 2.** Pattern analysis of the data from UPLC/TOF-MS analysis of serum samples from the sham and MI groups. (A) PCA score plot (R^2^X = 0.693, Q^2^ = 0.542) indicates the first two principal components: the sham samples are marked in green circles, and the MI samples are marked in blue circles; (B) OPLS-DA score plot (R^2^X = 0.491, R^2^Y = 0.99, Q^2^ = 0.978; CV-ANOVA *p*-value = 5.5687 E^-38^) indicates one predictive component and one orthogonal component: the sham samples are marked in green circles, and the MI samples are marked in blue circles; (C) A combination plot of *S*-plot and VIP values; p (corr) values are marked in purple circles, VIP values are marked in black squares.








**Supplementary Figure 3.** Univariate ROC curve analysis for individual metabolic biomarkers of MI.


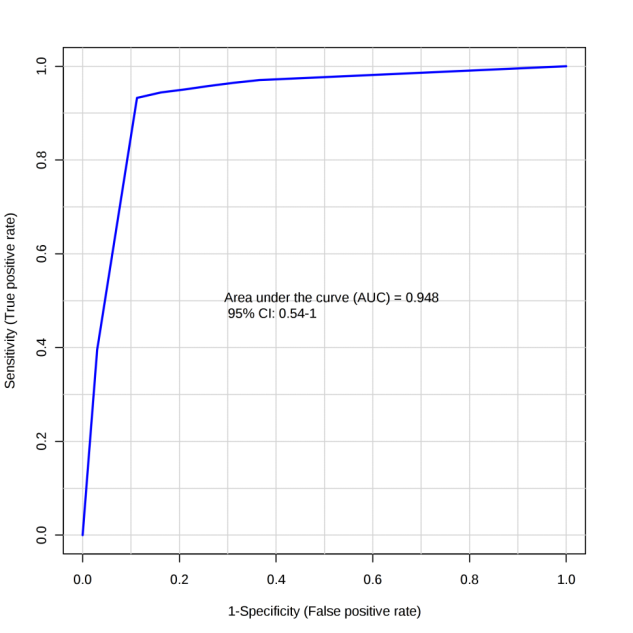

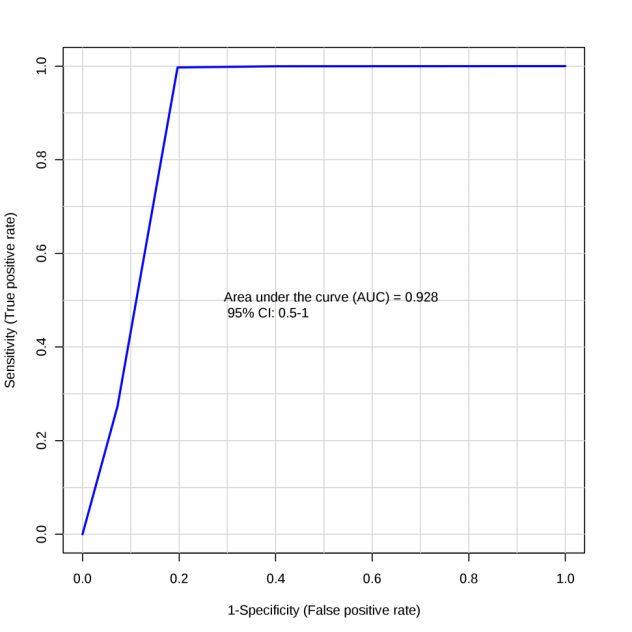


**B**

**A**


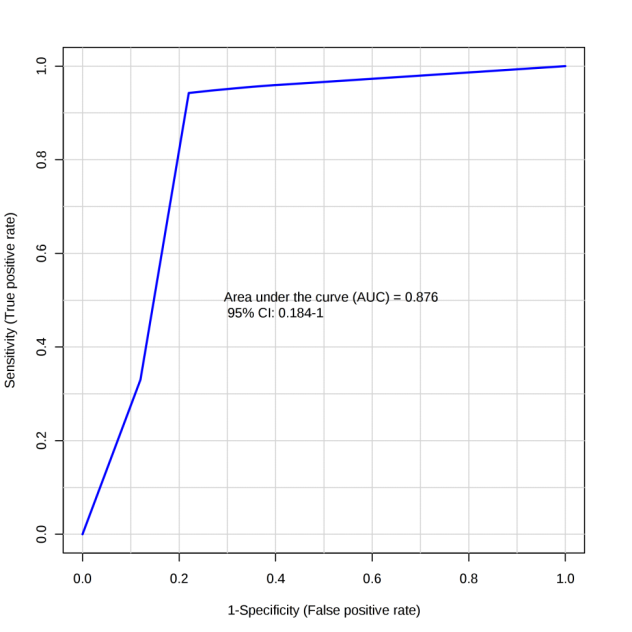

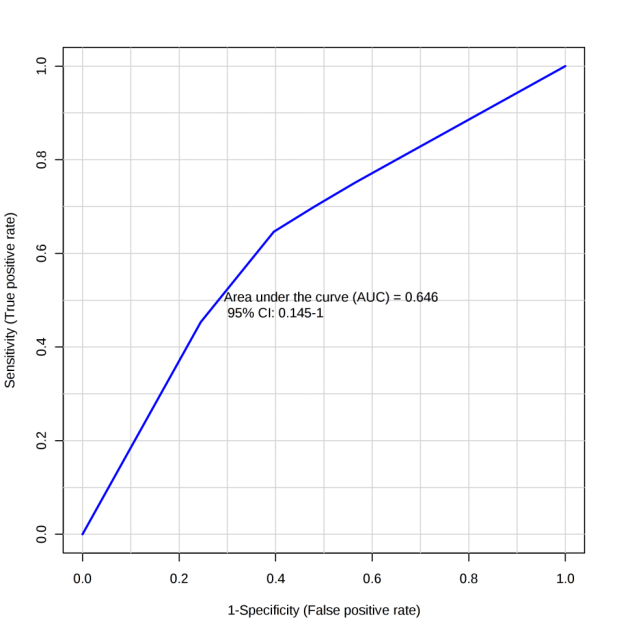


**D**

**C**

**Supplementary Figure 4.** Receiver operating characteristic (ROC) curve-based model evaluation. (A) MI group vs. sham group; (B) High dose group of BYD-treated vs. MI group; (C) Middle dose group of BYD-treated vs. MI group; (D) Low dose group of BYD-treated vs. MI group.


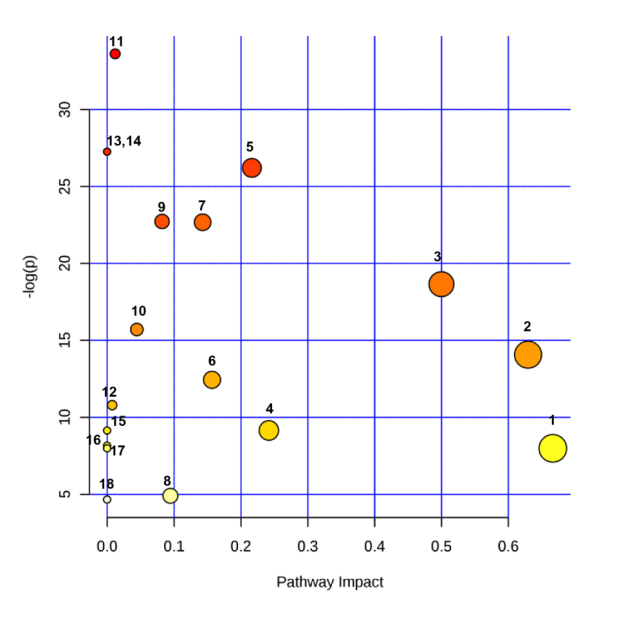

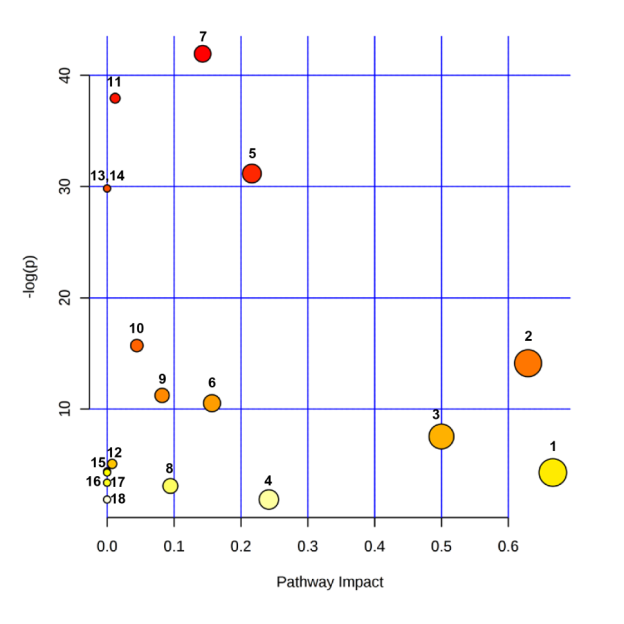


**A**

**B**


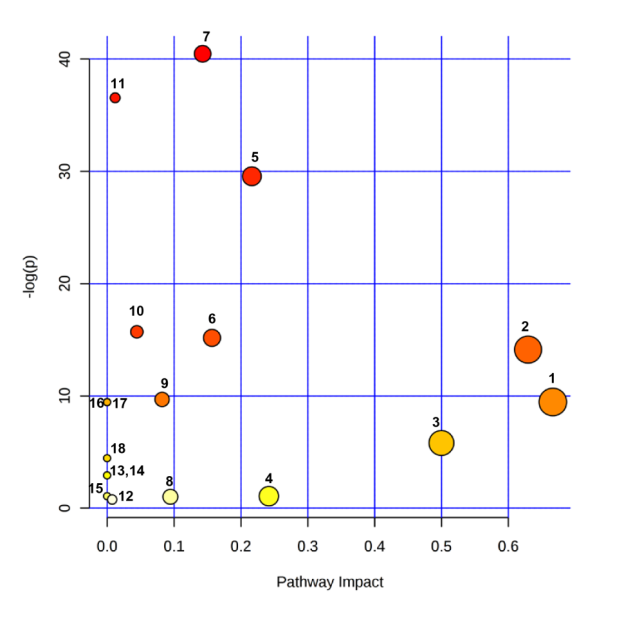

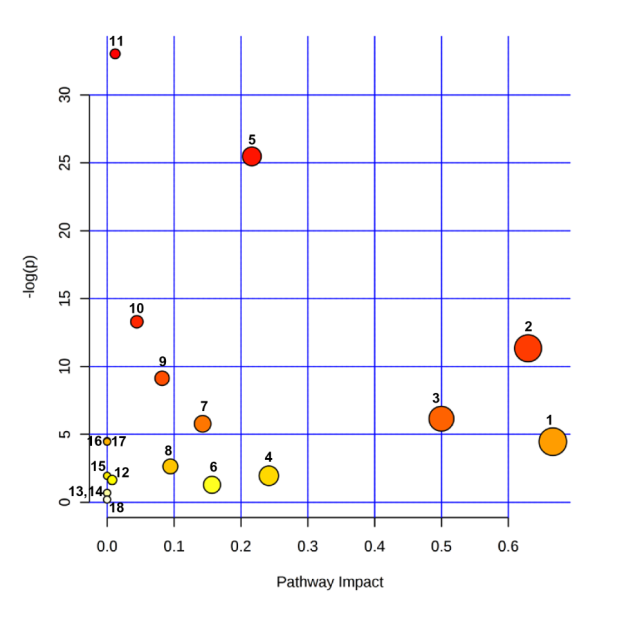


**D**

**C**

**Supplementary Figure 5.** Summary of the metabolic pathway enrichment analysis in MetaboAnalyst. (A) MI target metabolic pathway alterations; (B) Pathways influenced by the high dose of BYD; (C) Pathways influenced by the middle dose of BYD; (D) Pathways influenced by the low dose of BYD. Interpretation: 1. Valine, leucine and isoleucine biosynthesis; 2. Phenylalanine metabolism; 3. Phenylalanine, tyrosine and tryptophan biosynthesis; 4. Histidine metabolism; 5. Retinol metabolism; 6. Tryptophan metabolism; 7. Sphingolipid metabolism; 8. Cysteine and methionine metabolism; 9, Arginine and proline metabolism; 10. Glycerophospholipid metabolism; 11. Pyrimidine metabolism; 12. Purine metabolism; 13. Biosynthesis of unsaturated fatty acids; 14. Fatty acid biosynthesis; 15. Nitrogen metabolism; 16. Aminoacyl-tRNA biosynthesis; 17. Valine, leucine and isoleucine degradation; 18. Pantothenate and CoA biosynthesis.

**A**

**B**

**Supplementary Figure 6.** Pattern analysis of the data from the transcriptional profiles of myocardial tissues. (A) PCA score plot (R^2^X = 0.884, Q^2^ = 0.676) indicates the first two principal components; (B) OPLS-DA score plot (R^2^X =0.879, R^2^Y = 0.998, Q^2^ = 0.978; CV-ANOVA *p*-value = 0.00033) indicates one predictive component and one orthogonal component. Sham samples are marked in green circles, while MI samples are in blue circles.


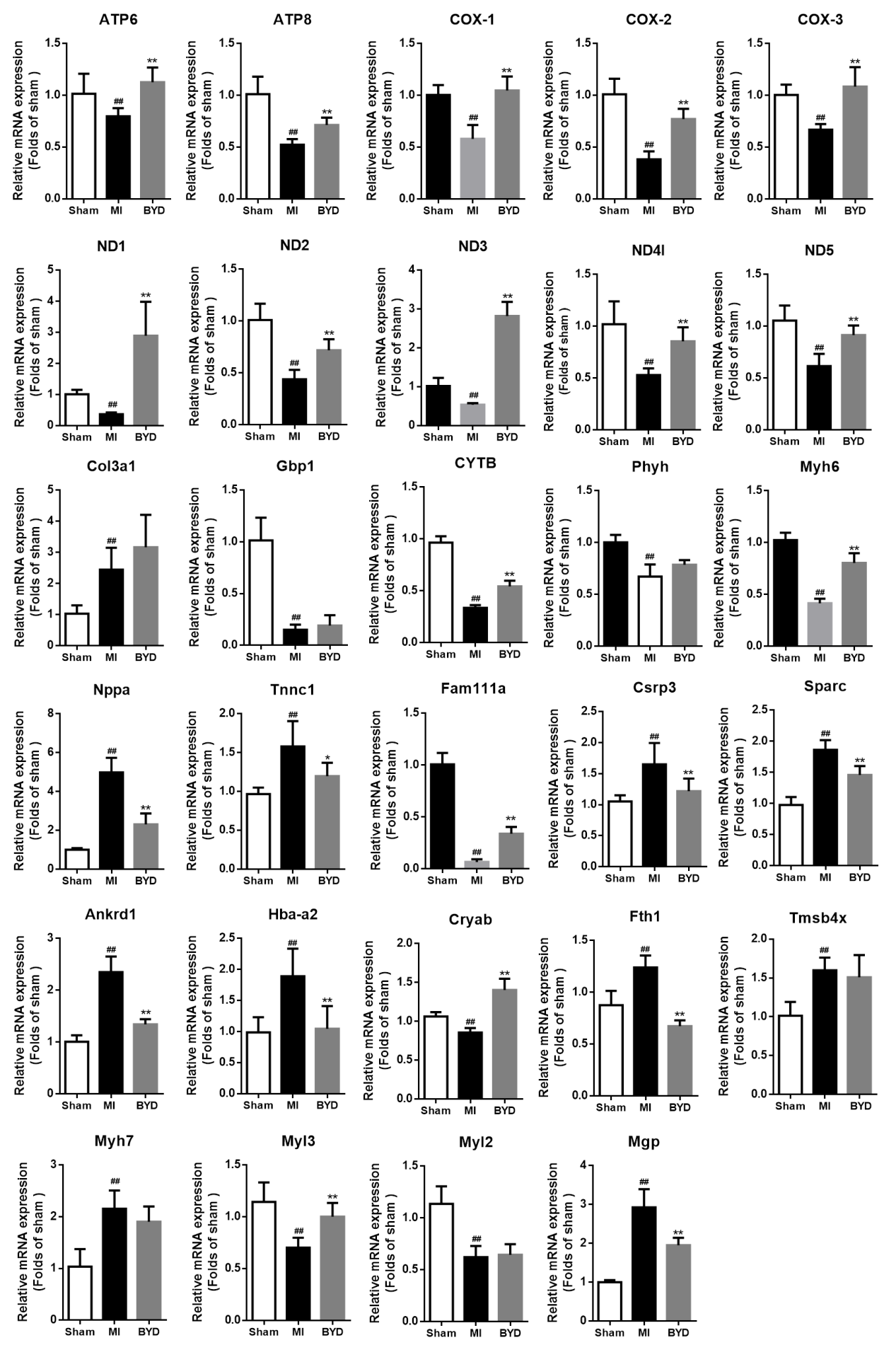


**Supplementary Figure 7.** Effects of BYD on the mRNA expressions of the significantly altered genes in MI rat heart tissues by qRT-PCR assay. The expression of each target was normalized to that of the Gapdh. Values are presented as means ± SD. Statistical analysis was performed using the one-way ANOVA (^##^*p*< 0.01 when MI compared to Sham ; **p*< 0.05 and ***p*< 0.01 when BYD compared to MI).


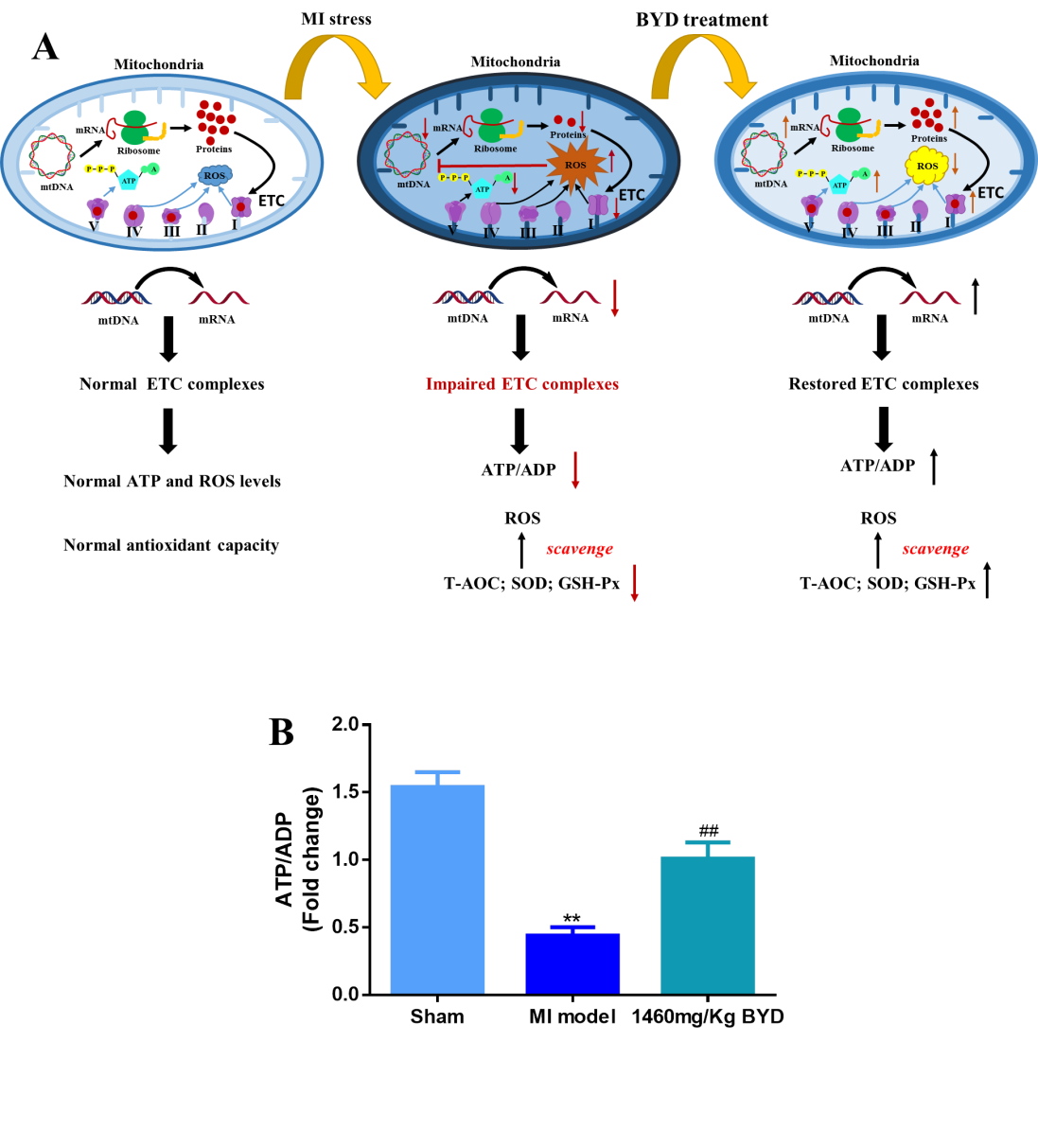


**Supplementary Figure 8.** Effects of BYD on energy metabolism in the rat myocardium subjected to MI. (A) Schematic illustrations of the effects of BYD on the transcript expressions that encode ETC complexes. (B) Effects of BYD on the ratio of ATP/ADP in the rat myocardium. Values are presented as means ± SD. Statistical analysis was performed using the one-way ANOVA (***p*< 0.01 when MI compared to Sham; ^##^*p*< 0.01 when BYD compared to MI).


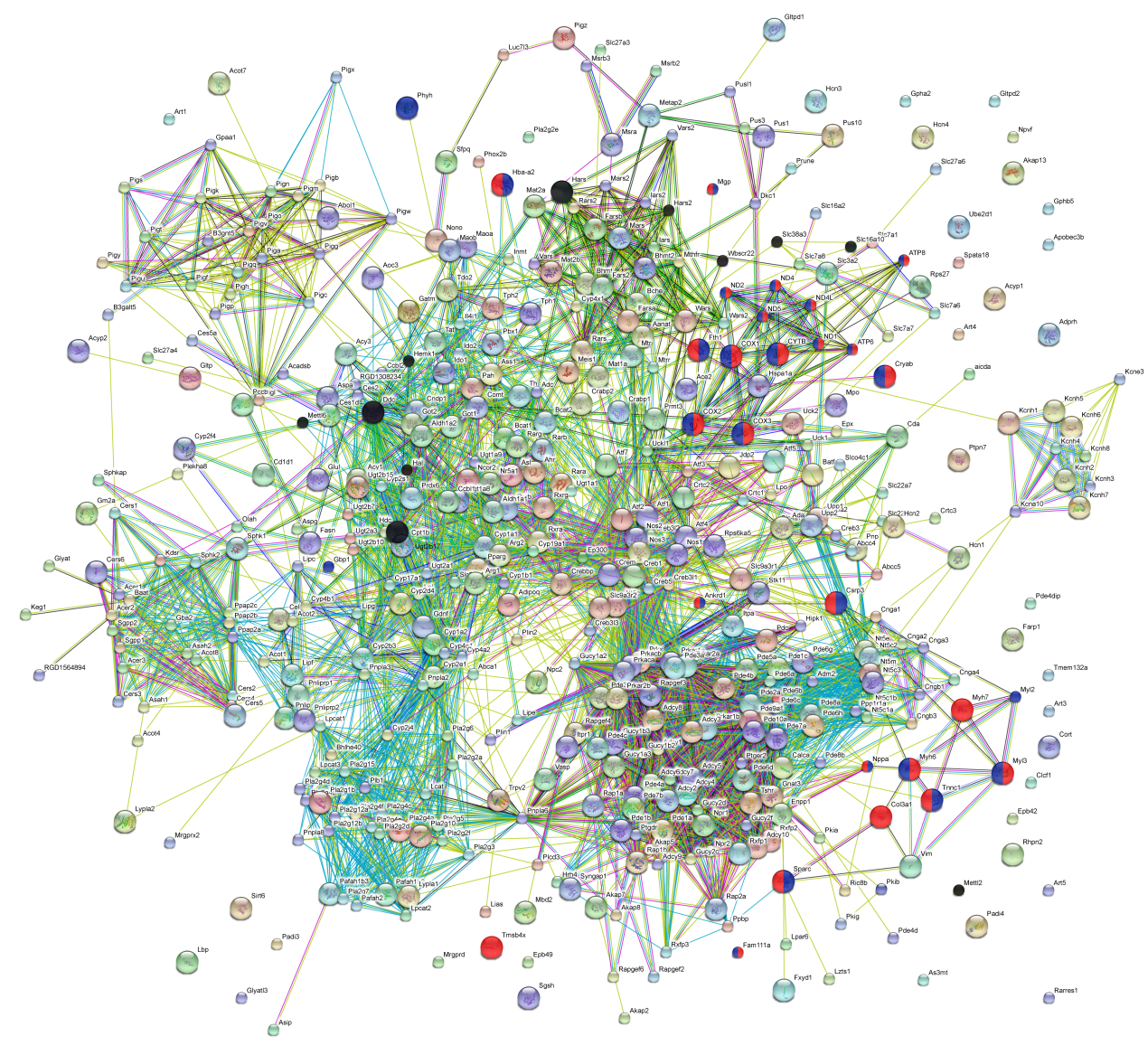


**Supplementary Figure 9.** Interaction analysis of metabolic biomarkers related- proteins and differentially expressed transcripts. The scarlet represents “upregulation”; the dark blue represents “downregulation”. The differentially expressed transcripts in the MI rats compared with the sham control are marked in the left semicircle, and those altered transcripts which can be regulated by the high dose of BYD compared with the MI rats are marked in the right semicircle; In addition, the non BYD-target candidate transcripts are marked in a full circle, and the non BYD-target metabolic biomarkers related proteins are marked in a full black circle.


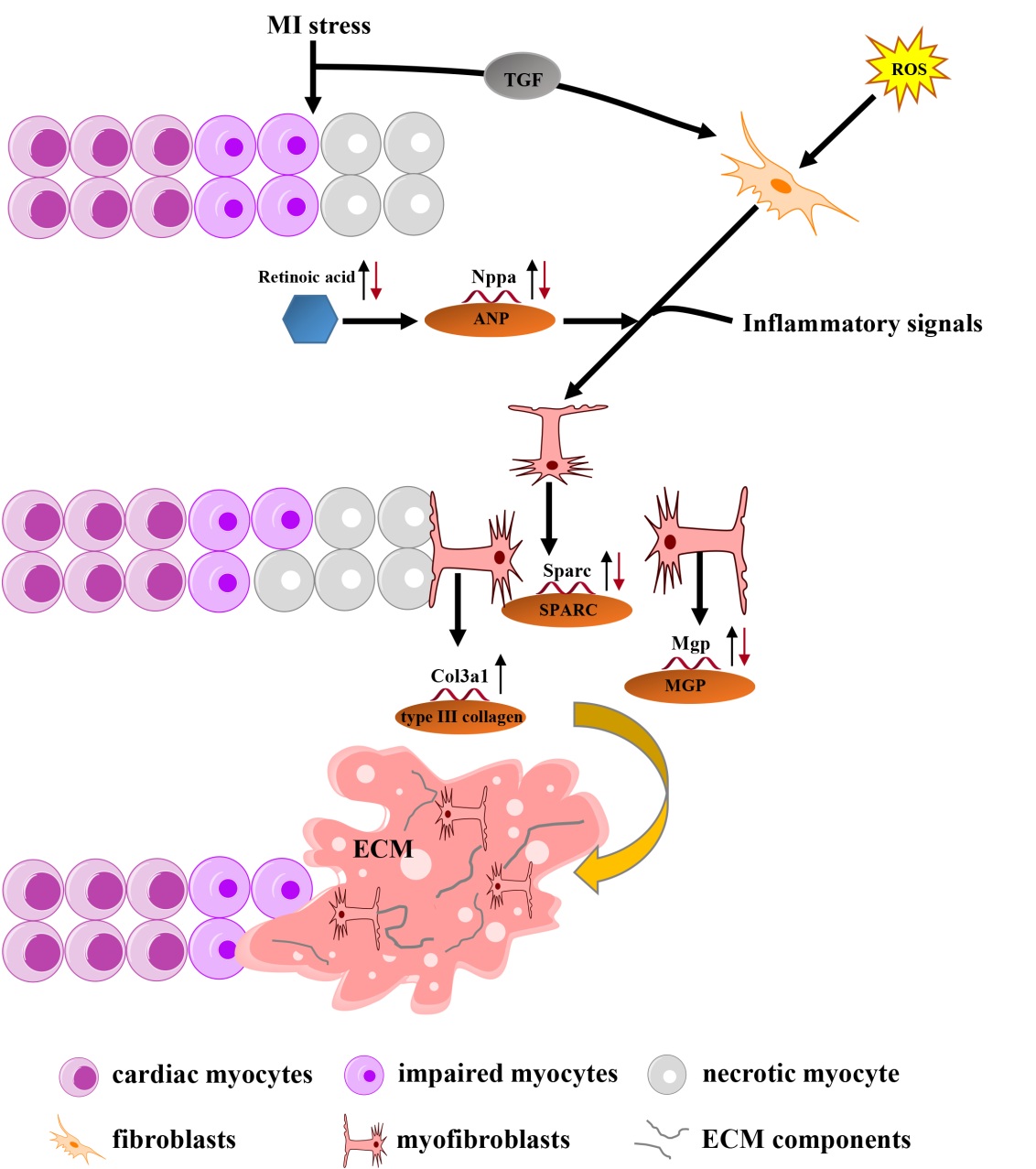


**Supplementary Figure 10.** Effects of BYD on extracellular matrix remodeling after MI. BYD leads to decreased expressions of Sparc, Mgp, and Nppa transcripts and retinoic acid in the MI rats, which indicates that BYD can attenuate the pathological fibrosis by mediating ECM remodeling after MI. The left black arrow “↑” or “↓”respectively indicates a decreased or increased level of the transcript or metabolite in the MI group compared with the sham group; The right red arrow “↓” or “↑”respectively indicates the decreased or increased, level of the transcript or metabolite in the high dose of BYD-treated group compared with the MI group. Abbreviations: transforming growth factor, TGF.

**1.2 Supplementary Tables**

**Supplementary Table 1. DNA oligonucleotide primer sequences used for qRT-PCR analysis of differentially expressed genes**

| No. | Gene symbol | GenBank ID | Description | Forward | Reverse | Amplicon length |
| --- | --- | --- | --- | --- | --- | --- |
| 1 | ATP8 | 26196 | Mitochondrially encoded ATP synthase 8 | TGCCACAACTAGACACATCCA | TAGTTTTGGGGGAGGGAGGT | 123 |
| 2 | ATP6 | 26197 | Mitochondrially encoded ATP synthase 6 | AACGCCTAATCAGCAACCGA | GGGCTCAGGTTCGTCCTTTT | 105 |
| 3 | COX2 | 26198 | Mitochondrially encoded cytochrome c oxidase II | ACAAGCACAATAGACGCCCA | ATTCGTAGGGAGGGAAGGGC | 92 |
| 4 | COX3 | 26204 | Mitochondrially encoded cytochrome c oxidase III | GAACATACCAAGGCCACCAC | TGGGTAGGAACTAGGCTGGAA | 136 |
| 5 | COX1 | 26195 | Mitochondrially encoded cytochrome c oxidase 1 | AGCTTTTGACTGCTTCCTCCA | GCTAGGTTTCCGGCTAAGGG | 110 |
| 6 | ND1 | 26193 | Mitochondrially encoded NADH dehydrogenase 1 | CCGCCTGACCAATAGCCATA | AATGGTCCTGCGGCGTATTC | 130 |
| 7 | ND2 | 26194 | Mitochondrially encoded NADH dehydrogenase 2 | TCCTCATAGGGCCTGTAATCA | GTTGGCTAGAAGTGGGATGATAG | 101 |
| 8 | ND4 | 26201 | Mitochondrially encoded NADH dehydrogenase 4 | CCTTCCCACACACGAGAATTA | TGTTAGGCCTGTGATGAGTTTAG | 93 |
| 9 | ND4l | 26200 | Mitochondrially encoded NADH dehydrogenase 4l | ACTCTCCTCTGCCTAGAAGGAA | AAACCTACTGCTGCTTCGCA | 140 |
| 10 | ND5 | 26202 | Mitochondrially encoded NADH dehydrogenase5 | AGCAATCTGTGCTCTCACCC | GCATGGGTGCAAATGTGGAG | 135 |
| 11 | Myh6 | 29556 | Myosin, heavy chain 6, cardiac muscle, alpha | CCATCCTCATCACTGGAGAATC | GGTGCCCTTGTTTGCATTAG | 131 |
| 12 | CYTB | 26192 | Mitochondrially encoded cytochrome b | CCTTCCTACCATTCCTGCATAC | TGGCCTCCGATTCATGTTAAG | 118 |
| 13 | Fam111a | 499322 | Family with sequence similarity 111, member A | TTAAACCTTGGCATGCCCCT | TGCTTGGTCCTGTGGTTCAA | 120 |
| 14 | Gbp1 | 304266 | Guanylate binding protein 1 | GGAGTGCAAAGCCAAAACCC | CTGCCGATGGCACTGAGAT | 93 |
| 15 | Phyh | 114209 | Phytanoyl-CoA 2-hydroxylase | ATATCAGCGTGAAGGGCACC | CCTTTCACAAGTCGGCATCG | 133 |
| 16 | Myl3 | 24585 | Myosin, light chain 3, alkali; ventricular, skeletal, slow | AAGATCACGTACGGGCAGTG | GAGCTCTTCCTGTTTGGGCT | 105 |
| 17 | Myl2 | 363925 | Myosin, light chain 2, regulatory, cardiac, slow | TGTTTGAGCAGACCCAGATCC | ATCTCTCGACTCTCTTCCCCG | 105 |
| 18 | Cryab | 25420 | Crystallin, alpha B | GCACCTGTTGGAGTCTGACC | AGAGCCCAGTGTCAATCCAG | 107 |
| 19 | Csrp3 | 117505 | Cysteine and glycine-rich protein 3 (cardiac LIM protein) | AGCCTGGAGTCCACAAATG | TGTGTAAGCCCTCCAAACC | 107 |
| 20 | Mgp | 25333 | Matrix Gla protein | AGTCCGGGAACTCAACAAGC | GGCGTTGTACCCGTAGATCA | 100 |
| 21 | Myh7 | 29557 | Myosin, heavy chain 7, cardiac muscle, beta | TGCTGGCACCGTGGACTA | CAGATTACTTAGGAGCTTGAGG | 104 |
| 22 | Ankrd1 | 27064 | Ankyrin repeat domain 1 | TAGAGGAGCTGGTAACGGGC | TCACACTGTTGGCTGGAAGT | 144 |
| 23 | Nppa | 24602 | Natriuretic peptide A | CTGGGACCCCTCCGATAGAT | GTCAATCCTACCCCCGAAGC | 106 |
| 24 | Sparc | 24791 | Secreted protein, acidic, cysteine-rich (osteonectin) | CTGGCAGCCCCTCAGAC | TCGACAGTTTCCTCTGCACC | 140 |
| 25 | Col3a1 | 84032 | Collagen, type III, alpha 1 | CAGGCCAATGGCAATGTAAAG | GCCATCCTCTAGAACTGTGTAAG | 108 |
| 26 | Tmsb4x | 81814 | Thymosin beta 4 | TGACAAACCCGATATGGCTGA | GGCAGAGGATTTTTCTCTTGTGT | 84 |
| 27 | Fth1 | 25319 | Ferritin, heavy polypeptide 1 | TGTATGCCTCCTACGTCTATCT | CTCAGCATGTTCCCTCTCTTC | 119 |
| 28 | Hba-a2 | 25632 | Hemoglobin alpha, adult chain 2 | AAACTGCGTGTGGATCCTGT | CGGGTGTGAAATCTCCAGGG | 91 |
| 29 | Tnnc1 | 290561 | Troponin C type 1 (slow) | CACAGGTGAGACCATCACGG | ACGCCCTTCATGAACTCCAG | 111 |
| 30 | Tnni3 | 29248 | Troponin I3, cardiac type | CACCTCAAGCAGGTGAAGAA | TCTTTCGGCCTTCCATTCC | 103 |
| 31 | Tnnt2 | 24837 | Troponin T2, cardiac type | CAAGGAGCTATGGCAGAGTATC | CGCAGAACGTTGATTTCGTATT | 96 |
| 32 | Mt-nd6 | 26203 | Mitochondrially encoded NADH dehydrogenase 6 | AGTGGATGTATTGGGTGCTTAAT | GGGTACTCCTCAGTAGCCATAG | 134 |
| 33 | Acta1 | 29437 | Actin, alpha 1 | CGCCAACAACGTCATGTCAG | CCACACTGAGTACTTGCGCT | 139 |
| 34 | Actc1 | 29275 | Actin, alpha, cardiac muscle 1 | CTTTGTCACCACCGCTGAAC | GGCCATCGGGAAGTTCGTAG | 140 |
| 35 | Fgf12 | 170630 | Fibroblast growth factor 12 | CCCCAGCTGAAAGGGATTGT | TCGCTGTTTTCGTCCTTGGT | 104 |
| 36 | Gapdh | 24383 | Glyceraldehyde-3-phosphate dehydrogenase | TCTCTTGTGACAAAGTGGACAT | AACTTGCCGTGGGTAGAGTC | 100 |

**Supplementary Table 2. Identification of significantly differential metabolites in rat serum.**

| No. | R.T. (min) | Exact mass (*m/z*) | Daughter ions (*m/z*) | Formula | Identification | MI vs. Sham | HBYD vs. MI | MBYD vs. MI | LBYD vs. MI |
| --- | --- | --- | --- | --- | --- | --- | --- | --- | --- |
| 1 | 3.5643 | 227.1270 | 110.0131; 156.1643 | C_9_H_14_N_4_O_3_ | Carnosine | **↑**^Δ^* | **↓**^Δ^* | **↓**^Δ^* | **↓**^Δ^* |
| 2 | 6.2298 | 253.1427 | 117.2316 | C_10_H_12_N_4_O_4_ | Deoxyinosine | **↑**^Δ^* | **↓**^Δ^* | **↓**^Δ^* | **↓**^Δ^* |
| 3 | 4.6368 | 245.0794 | 71.5608; 57.2091 | C_9_H_12_N_2_O_6_ | Uridine ^a^ | **↑**^Δ^* | **↓**^Δ^* | **↓**^Δ^* | **↓**^Δ^* |
| 4 | 5.9641 | 302.3067 | 279.0939; 224.1314 | C_18_H_39_NO_2_ | Sphinganine ^a^ | **↓**^Δ^* | **↑**^Δ^* | **↑**^Δ^* | **↑**^Δ^* |
| 5 | 6.2343 | 175.0981 | 70.0661; 116.0710 | C_6_H_14_N_4_O_2_ | L-Arginine ^a^ | **↑**^Δ^* | **↓**^Δ^* | **↓**^Δ^* | **↓**^Δ^* |
| 6 | 1.5158 | 346.2642 | 152.1331 | C_10_H_12_N_5_O_7_P | Cyclic GMP ^a^ | **↑**^Δ^* | **↓**^Δ^* | **↓**^Δ^* | - |
| 7 | 6.7354 | 260.0354 | 161.9931 | C_6_H_13_NO_8_S | N-Sulfo-D-glucosamine | **↑**^Δ^* | **↓**^Δ^* | **↓**^Δ^* | **↓**^Δ^* |
| 8 | 7.3600 | 285.2230 | 135.901; 109.9451 | C_10_H_12_N_4_O_6_ | Xanthosine | **↓**^Δ^* | **↑**^Δ^* | **-** | **-** |
| 9 | 4.1202 | 180.1379 | 135.0913; 147.0991 | C_9_H_9_NO_3_ | Hippuric acid | **↑**^Δ^* | **↓**^Δ^* | **↓**^Δ^* | **↓**^Δ^* |
| 10 | 7.5565 | 301.1424 | 135.1103 | C_20_H_28_O_2_ | Retinoic acid ^a^ | **↑**^Δ^* | **↓**^Δ^* | **↓**^Δ^* | **↓**^Δ^* |
| 11 | 7.6642 | 149.0248 | 59.0121 | C_4_H_4_O_6_ | Dihydroxyfumaric acid | **↑**^Δ^* | **↓**^Δ^* | **↓**^Δ^* | - |
| 12 | 7.6609 | 205.0872 | 188.0811 | C_11_H_12_N_2_O_2_ | L-Tryptophan ^a^ | **↑**^Δ^* | **↓**^Δ^* | **↓**^Δ^* | - |
| 13 | 7.3131 | 330.3377 | 136.0612 | C_10_H_12_N_5_O_6_P | Cyclic AMP ^a^ | **↑**^Δ^* | **↓**^Δ^* | **↓**^Δ^* | **↓**^Δ^* |
| 14 | 0.7617 | 132.1033 | 112.0161; 111.018 | C_6_H_13_NO_2_ | L-Isoleucine ^a^ | **↑**^Δ^* | **↓**^Δ^* | **↓**^Δ^* | **↓*** |
| 15 | 0.7669 | 122.0579 | 105.0214; 51.0235 | C_8_H_11_N | Phenylethylamine ^a^ | **↑**^Δ^* | **↓**^Δ^* | **↓**^Δ^* | **↓**^Δ^* |
| 16 | 2.3654 | 156.0421 | 110.0381 | C_6_H_9_N_3_O_2_ | L-Histidine ^a^ | **↓**^Δ^* | **-** | **-** | - |
| 17 | 0.5551 | 150.0600 | 103.9971 | C_5_H_11_NO_2_S | L-Methionine ^a^ | **↓**^Δ^* | **↑**^Δ^* | **-** | - |
| 18 | 9.9847 | 524.3711 | 184.0735 | C_26_H_54_NO_7_P | LysoPC(18:0) ^a^ | **↑**^Δ^* | **↓**^Δ^* | **↓**^Δ^* | **↓**^Δ^* |
| 19 | 8.3618 | 510.3546 | 184.0739 | C_25_H_52_NO_7_P | LysoPC(17:0) ^a^ | **↑**^Δ^* | **↓**^Δ^* | **↓**^Δ^* | - |
| 20 | 4.8704 | 285.2915 | 263.2164 | C_18_H_36_O_2_ | Stearic acid ^a^ | **↑**^Δ^* | **↓**^Δ^* | **-** | - |
| 21 | 3.2213 | 166.0247 | 121.0637 | C_9_H_11_NO_2_ | L-Phenylalanine ^a^ | **↑**^Δ^* | **↓**^Δ^* | **↓**^Δ^* | **↓**^Δ^* |
| 22 | 0.9464 | 118.0881 | 72.9131 | C_5_H_11_NO_2_ | L-Valine ^a^ | **↓**^Δ^* | **↑**^Δ^* | **↑**^Δ^* | - |

Notes: “Δ” indicates AUC area value ≥ 0.8; “*” indicates the Student’s *t*-test value of *p* < 0.05; “–” indicates no statistically significant difference; Arrow “↑” or “↓” indicates an increased or decreased level of metabolite; “a” indicates metabolites confirmed with authentic standards. HBYD, MBYD, and LBYD represent the high, middle, and low dose of BYD-treated groups, respectively.

**Supplementary Table 3. Results from ingenuity pathway analysis with MetPA**

| No. | Pathway Name | Total | Hits | Impact | Sham versus MI | | HBYD versus MI | | MBYD versus MI | | LBYD versus MI | |
| --- | --- | --- | --- | --- | --- | --- | --- | --- | --- | --- | --- | --- |
|  |  |  |  |  | *p* | FDR | *p* | FDR | *p* | FDR | *p* | FDR |
| 1 | Valine, leucine and isoleucine biosynthesis | 11 | 2 | 0.6667 | 3.41E-04 | 3.84E-04 | 0.013666 | 0.01757 | 7.83E-05 | 1.43E-04 | 0.01152 | 0.02073 |
| 2 | Phenylalanine metabolism | 9 | 2 | 0.6296 | 7.75E-07 | 1.55E-06 | 7.36E-07 | 1.89E-06 | 7.36E-07 | 2.21E-06 | 1.19E-05 | 5.34E-05 |
| 3 | Phenylalanine, tyrosine and tryptophan biosynthesis | 4 | 1 | 0.5 | 7.93E-09 | 2.04E-08 | 5.36E-04 | 9.65E-04 | 0.003018 | 0.004939 | 0.002126 | 0.006377 |
| 4 | Histidine metabolism | 15 | 2 | 0.2419 | 1.07E-04 | 1.48E-04 | 0.15479^▼^ | 0.15491 | 0.34481^▼^ | 0.38598 | 0.14155^▼^ | 0.197 |
| 5 | Retinol metabolism | 17 | 1 | 0.2165 | 4.14E-12 | 1.86E-11 | 2.90E-14 | 1.74E-13 | 1.45E-13 | 8.68E-13 | 8.70E-12 | 7.83E-11 |
| 6 | Tryptophan metabolism | 41 | 1 | 0.1568 | 3.97E-06 | 7.15E-06 | 2.68E-05 | 5.37E-05 | 2.58E-07 | 9.28E-07 | 1.08E-04 | 3.89E-04 |
| 7 | Sphingolipid metabolism | 21 | 1 | 0.1429 | 1.42E-10 | 4.26E-10 | 6.12E-19 | 1.10E-17 | 2.64E-18 | 4.75E-17 | 0.003089 | 0.007942 |
| 8 | Cysteine and methionine metabolism | 28 | 1 | 0.09464 | 0.007462 | 0.007901 | 0.045902 | 0.05164 | 0.36453^▼^ | 0.386 | 0.07126^▼^ | 0.11661 |
| 9 | Arginine and proline metabolism | 44 | 1 | 0.08228 | 1.35E-10 | 4.26E-10 | 1.34E-05 | 3.01E-05 | 6.23E-05 | 1.43E-04 | 1.08E-04 | 3.89E-04 |
| 10 | Glycerophospholipid metabolism | 30 | 1 | 0.04444 | 1.51E-07 | 3.40E-07 | 1.51E-07 | 4.53E-07 | 1.51E-07 | 6.79E-07 | 1.68E-06 | 1.01E-05 |
| 11 | Pyrimidine metabolism | 41 | 1 | 0.01202 | 2.50E-15 | 4.50E-14 | 3.32E-17 | 2.99E-16 | 1.33E-16 | 1.20E-15 | 4.57E-15 | 8.22E-14 |
| 12 | Purine metabolism | 68 | 4 | 0.00755 | 2.06E-05 | 3.37E-05 | 0.006278 | 0.01027 | 0.46598^▼^ | 0.46598 | 0.19353^▼^ | 0.2488 |
| 13 | Biosynthesis of unsaturated fatty acids | 42 | 1 | 0 | 1.45E-12 | 8.69E-12 | 1.11E-13 | 3.98E-13 | 0.05407^▼^ | 0.069522 | 0.50061^▼^ | 0.53006 |
| 14 | Fatty acid biosynthesis | 43 | 1 | 0 | 1.45E-12 | 8.69E-12 | 1.11E-13 | 3.98E-13 | 0.05407^▼^ | 0.069522 | 0.5006^1▼^ | 0.53006 |
| 15 | Nitrogen metabolism | 9 | 1 | 0 | 1.07E-04 | 1.48E-04 | 0.15491^▼^ | 0.15491 | 0.34539^▼^ | 0.38598 | 0.14231^▼^ | 0.197 |
| 16 | Aminoacyl-tRNA biosynthesis | 67 | 7 | 0 | 2.86E-04 | 3.68E-04 | 0.012299 | 0.01757 | 7.97E-05 | 1.43E-04 | 0.01103 | 0.02073 |
| 17 | Valine, leucine and isoleucine degradation | 38 | 2 | 0 | 3.41E-04 | 3.84E-04 | 0.013666 | 0.01757 | 7.83E-05 | 1.43E-04 | 0.01152 | 0.02073 |
| 18 | Pantothenate and CoA biosynthesis | 15 | 1 | 0 | 0.009495 | 0.009495 | 0.034148 | 0.04098 | 0.01169 | 0.01754 | 0.81528^▼^ | 0.81528 |

Notes: Total is the total number of compounds in the pathway; Hits is the actually matched number from the user uploaded data; Impact is the pathway impact value calculated from the pathway topology analysis; *p* value calculated from the pathway enrichment analysis, “^▼^”indicates no statistically significant difference; FDR is the *p* value adjusted using the False Discovery Rate. HBYD, MBYD, and LBYD represent the high, middle, and low dose of BYD-treated groups, respectively.
